# HadD, a novel fatty acid synthase type II protein, is essential for alpha- and epoxy-mycolic acid biosynthesis and mycobacterial fitness

**DOI:** 10.1038/s41598-018-24380-5

**Published:** 2018-04-16

**Authors:** Cyril Lefebvre, Richard Boulon, Manuelle Ducoux, Sabine Gavalda, Françoise Laval, Stevie Jamet, Nathalie Eynard, Anne Lemassu, Kaymeuang Cam, Marie-Pierre Bousquet, Fabienne Bardou, Odile Burlet-Schiltz, Mamadou Daffé, Annaïk Quémard

**Affiliations:** 1 Département Tuberculose & Biologie des Infections, Institut de Pharmacologie et de Biologie Structurale, UMR5089, Université de Toulouse, CNRS, UPS, 31077 Toulouse Cedex 04, France; 2 Département Biologie Structurale & Biophysique, Institut de Pharmacologie et de Biologie Structurale, UMR5089, Université de Toulouse, CNRS, UPS, 31077 Toulouse Cedex 04, France

## Abstract

Mycolic acids (MAs) have a strategic location within the mycobacterial envelope, deeply influencing its architecture and permeability, and play a determinant role in the pathogenicity of mycobacteria. The fatty acid synthase type II (FAS-II) multienzyme system is involved in their biosynthesis. A combination of pull-downs and proteomics analyses led to the discovery of a mycobacterial protein, HadD, displaying highly specific interactions with the dehydratase HadAB of FAS-II. *In vitro* activity assays and homology modeling showed that HadD is, like HadAB, a hot dog folded (*R*)-specific hydratase/dehydratase. A *hadD* knockout mutant of *Mycobacterium smegmatis* produced only the medium-size alpha’-MAs. Data strongly suggest that HadD is involved in building the third meromycolic segment during the late FAS-II elongation cycles, leading to the synthesis of the full-size alpha- and epoxy-MAs. The change in the envelope composition induced by *hadD* inactivation strongly altered the bacterial fitness and capacities to aggregate, assemble into colonies or biofilms and spread by sliding motility, and conferred a hypersensitivity to the firstline antimycobacterial drug rifampicin. This showed that the cell surface properties and the envelope integrity were greatly affected. With the alarmingly increasing case number of nontuberculous mycobacterial diseases, HadD appears as an attractive target for drug development.

## Introduction

Mycobacteria are responsible for a large diversity of infectious diseases. The best known is tuberculosis (TB) since it represents one of the leading causes of death worldwide^1^. The emergence of multidrug and extensively drug-resistant *Mycobacterium tuberculosis* strains has challenged TB control^1^. The nontuberculous mycobacteria (NTM), such as *M*. *smegmatis*, *M*. *kansasii*, *M*. *abscessus*, and members of the *M*. *avium* and *M*. *fortuitum/chelonae* complexes, form a heterogeneous group of environmental organisms found in water, soil, dust, biofilms and diverse animals^2^. Pathogenic NTM can cause infectious diseases in livestock and wildlife, as well as human diseases, such as pulmonary and disseminated diseases, lymphadenitis, and skin and soft tissue lesions^2,3^. Like *M*. *tuberculosis*, they constitute a major cause of opportunistic infections in HIV patients^4^. Alarmingly, the number of reported cases has been increasing worldwide^2,5^. NTM also colonize man-made environments and cause healthcare-associated infections, which are a major public health concern^2–4^. NTM are intrinsically resistant to most conventionally utilized antimicrobials and many of the antituberculous drugs under development are inactive against NTM^4,5^. Thus, novel molecular strategies for eradicating NTM from infection sources and hosts are urgently needed^5^.

The very thick envelope of mycobacteria is characterized by a broad array of exotic complex lipids, such as glycopeptidolipids (GPLs), trehalose polyphleates (TPPs) and the mycolate-containing compounds, which are key players in the infectious processes of pathogenic species^6–8^. The mycolate-containing compounds are made of mycolic acids (MAs), unusually long α-alkylated β-hydroxylated fatty acids, esterifying either the polysaccharide arabinogalactan extremities or polyol molecules such as trehalose, forming the trehalose monomycolate (TMM) and the trehalose dimycolate (TDM)^9^. The anchoring of the mycolate-containing lipids in the outer membrane (or mycomembrane) confers them a strategic position within the envelope, greatly affecting its architecture and its permeability^7,10^. Large amounts of free MAs are also present in the extracellular matrix of mycobacterial biofilms^11,12^. The MA-containing compounds are essential to the survival of mycobacteria and crucial for their physiology and fitness^7^. Furthermore, they modulate the immune response to infection by pathogenic mycobacteria, thereby playing a role in their virulence and their persistence within the host^7,13,14^. MAs are the products of a mixed fatty acid synthase (FAS)/polyketide synthase biosynthesis pathway^9^, which represents a validated source of pharmacological targets^7,15^. MA subclasses typifying the different mycobacterial species are characterized by various types of chemical function (cyclopropane ring, double bond, methyl branch and oxygenated functions), at the proximal and distal positions of their main ‘meromycolic’ chain^7^, which modulate the biological properties of the molecules^7,13^. The biosynthetic filiation between the various types of MA is matter of debate. Yet, the enzymatic differentiation of the meromycolic chains is known to be achieved by a family of paralogous S-adenosylmethionine-dependent MA methyltransferases (MA-MTs)^7^, most likely using a mechanism of *C*-methylation of isolated *cis* double bonds via the formation of an intermediate carbonium ion, demonstrated in the case of the *trans*-ethylenic MAs of *M*. *smegmatis*^16^. While most steps of the MA biosynthesis pathway are well characterized^9^, the mechanisms by which the double bonds are introduced in their meromycolic chain are still under investigation^7^. An array of data suggests that at least some of the modifications are formed during the iterative elongation cycles catalyzed by the FAS type II (FAS-II) system leading to the formation of the meromycolic chains^17–19^. Furthermore, a recent survey suggests that the putative desaturase DesA1 might be involved in the introduction of one double bond at an as yet undefined position in the α-MAs of *M*. *smegmatis*^20^. However, further work is needed to establish a complete picture of the enzyme reactions governing the desaturation of all types of MA^7^.

In this context, we decided to prospect for unknown partner enzymes of the FAS-II system that could potentially introduce double bonds. We showed that MSMEG_0948 (HadD) protein from *M*. *smegmatis* interacts with the FAS-II heterodimeric dehydratase HadA-HadB (HadAB). With the aim of defining the role of HadD, its *in vitro* enzymatic activity, its structure and the impacts of its depletion on both the MA production and the physiology of mycobacteria were assessed.

## Results

### MSMEG_0948 protein specifically interacts with HadAB enzyme of the FAS-II system

**Figure 1:**
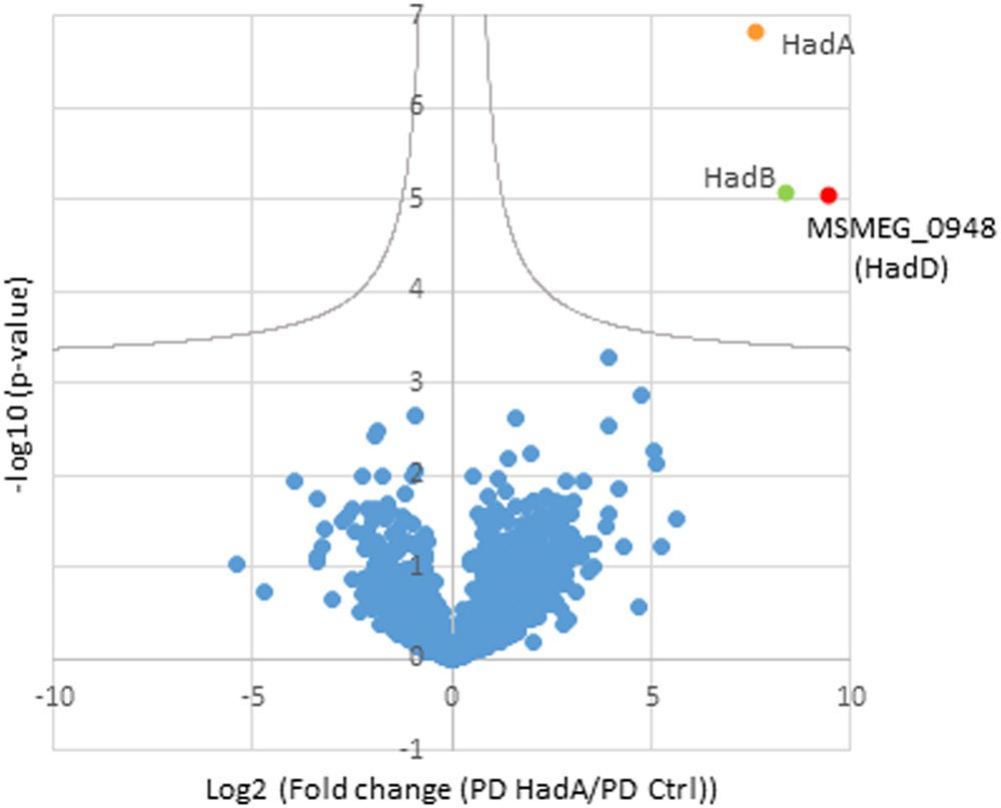
The dehydratase HadAB of FAS-II displays highly specific interaction with a novel protein, HadD (MSMEG_0948). Proteomics analysis of pull-down fractions using eGFP-HadA as a bait. Volcano plot [−log10 (p-value) versus log2 (fold change)] showing enriched proteins in eGFP-HadA-based pull-down (PD) versus control pull-down (using simple eGFP as a bait). Statistical analysis was performed from four independent biological replicates by two-sided Student t-test, variance correction and permutation-based false discovery rate (FDR) control in Perseus. Proteins considered as significantly enriched (FDR <5%, hyperbolic selection curve indicated in grey) are colored; the bait, HadA, is in orange, while copurifying proteins, HadB and HadD, are in green and red, respectively.

The (3 *R*)-hydroxyacyl-ACP dehydratases HadAB and HadBC are two main components of the mycobacterial FAS-II system, HadA and HadC being the substrate-binding subunits and HadB the catalytic subunit^21,22^. Accordingly, HadB is essential for the MA biosynthesis in *M*. *smegmatis* and *M*. *tuberculosis* and for the survival of *M*. *smegmatis*^21,23^. With the aim of discovering novel partner proteins of the FAS-II system putatively involved in double bond formation, pull-down experiments were performed from *M*. *smegmatis* bacterial lysates using HadA as a bait. For that, *hadABC* operon was expressed, with an enhanced Green Fluorescent Protein (eGFP) tag at the N-terminus of HadA, in a *M*. *smegmatis* strain deleted for the endogenous *hadABC* genes. The bait and interacting proteins were trapped using anti-GFP magnetic beads and, after extensive washes, released in mild conditions. The elution fractions, analyzed by nanoLC-MS/MS after trypsin digestion, displayed a strong and significant enrichment in HadB (Fig. 1), the second subunit of the HadAB enzyme. Strikingly, an unknown protein, MSMEG_0948, was observed at an equivalent enrichment level. This shows the occurrence of a highly specific physical interaction between HadAB enzyme and MSMEG_0948 protein.

### MSMEG_0948 is a hydratase/dehydratase

MSMEG_0948 has a theoretical mass of 19.6 kDa and is annotated as “conserved hypothetical protein” (http://mycobrowser.epfl.ch/smegmalist.html)^24^. It represents the closest mycobacterial homolog of HadA (41% identity) and HadC (35% identity) subunits of the FAS-II dehydratases HadAB and HadBC, while it is much more distant from HadB (Supplementary Fig. S1a). Structural homology modeling predicted that a MSMEG_0948 monomer would organize, like HadA, HadB and HadC, as a ‘single hot dog’ fold made of a central *α*-helix (*α3)* wrapped into a five-stranded antiparallel *β*-sheet (Supplementary Fig. S1b). Above this fold would sit a solvent-exposed loop corresponding to the “overhanging segment” first described in the (*R*)-hydratase from *Aeromonas caviae*^25^. This loop bears a degenerate hydratase 2 motif ‘F-x(2)-a-x(2)-**D**-x(2)-P-a-**H**-x(5)-A’, containing the catalytic dyad Asp (D34) and His (H39), at the same position as the hydratase 2 motif of HadB (Supplementary Fig. S1b). This strongly suggests that MSMEG_0948 protein is a (*R*)-specific hydratase/dehydratase related to the hydratase 2 subfamily, like HadAB and HadBC enzymes. We named it “HadD”.

**Figure 2:**
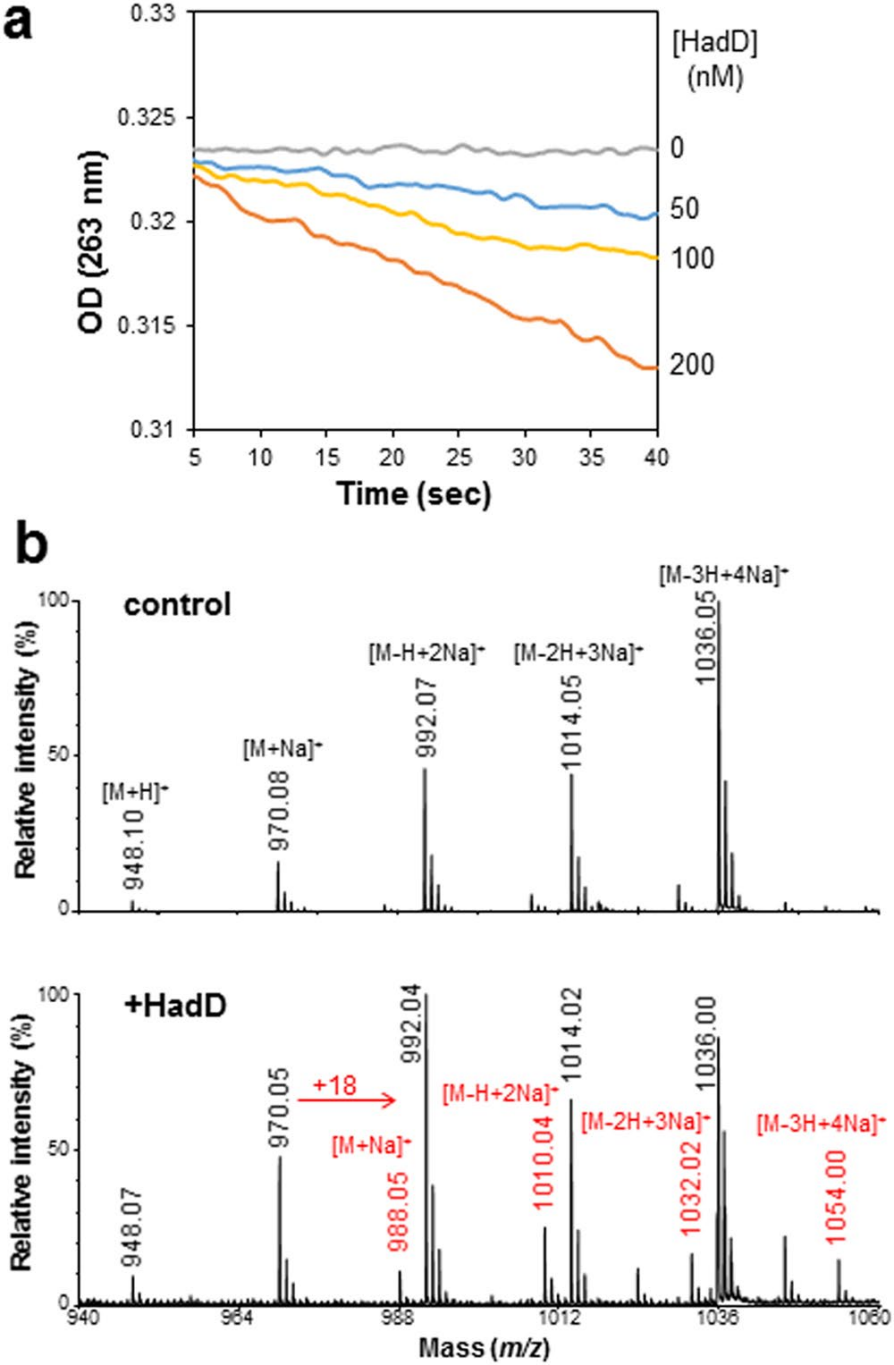
HadD belongs to the hydratase/dehydratase family. (**a**) Enzyme activity assays. Kinetic experiments were performed in 100 mM sodium phosphate buffer pH 7.0 in the presence of 10 µM C_12:1_-CoA and various concentrations of purified H-HadD protein, and monitored spectrophotometrically by following the disappearance of the *trans*-2-ethylenic function of the substrate at 263 nm. (**b**) MALDI-TOF MS analysis of the reaction product. Ions peaks of the hydration product, the 3-hydroxydodecanoyl-CoA, were detected in assays in the presence of HadD (bottom) but not in control assays without protein (top). The substrate ion peaks (proton and mono- to tetra-sodium adducts) are labeled in black and the hydration product ion peaks (mono- to tetra-sodium adducts) are labeled in red.

*MSMEG_0948* gene was cloned into pET-15b vector, and the N-terminal poly-His tagged protein (H-HadD) was produced in *Escherichia coli* and purified by cobalt-based immobilized metal affinity chromatography (Supplementary Fig. S2). Enzymes belonging to the (*R*)-specific enoyl hydratase/hydroxyacyl dehydratase family preferentially catalyze the hydration reaction when they are isolated from their enzymatic complex^21,26,27^. Thus, their activities are most often studied in the presence of enoyl derivatives *in vitro*. Kinetic activity assays were performed by spectrophotometry in the presence of purified H-HadD and *trans*-2-dodecenoyl-CoA, which can be used as a substrate by both HadAB and HadBC dehydratases^21^. Data showed that H-HadD protein was active and able to metabolize the *trans*-2-ethylenic function of the substrate (Fig. 2a). Increasing initial velocities were obtained in the presence of increasing H-HadD concentrations. The reaction led to an increment of 18 mass units, as shown by MALDI-TOF MS (Fig. 2b), corresponding to the hydration of the substrate into 3-hydroxydodecanoyl-CoA.

Bioinformatics analyses by BlastP searches^28^ using MSMEG_0948 protein sequence as a probe indicated that HadD is ubiquitous among all of the sequenced mycobacterial genomes. Interestingly, there is no MSMEG_0948 ortholog in *Corynebacterium* genus that synthesizes short chain MAs and has no FAS-II system. Most importantly, it is also absent from the other genera of the *Corynebacteriales* order, such as *Nocardia*, *Rhodococcus*, and *Gordonia*, which produce medium-chain mycolic acids. This is in sharp contrast with HadAB enzyme involved in early elongation steps, which is well conserved in these genera, and is reminiscent from the behavior of HadC subunit that is involved in late elongation steps^14,21^.

These data altogether demonstrated that HadD is a hydratase/dehydratase belonging to the FAS-II system and suggested that it might have a role in the late elongation steps required for the formation of the full-size meromycolic chains found in mycobacteria.

### Mutation of *hadD* abolishes the production of cell-wall linked α- and epoxy-MAs

**Figure 3:**
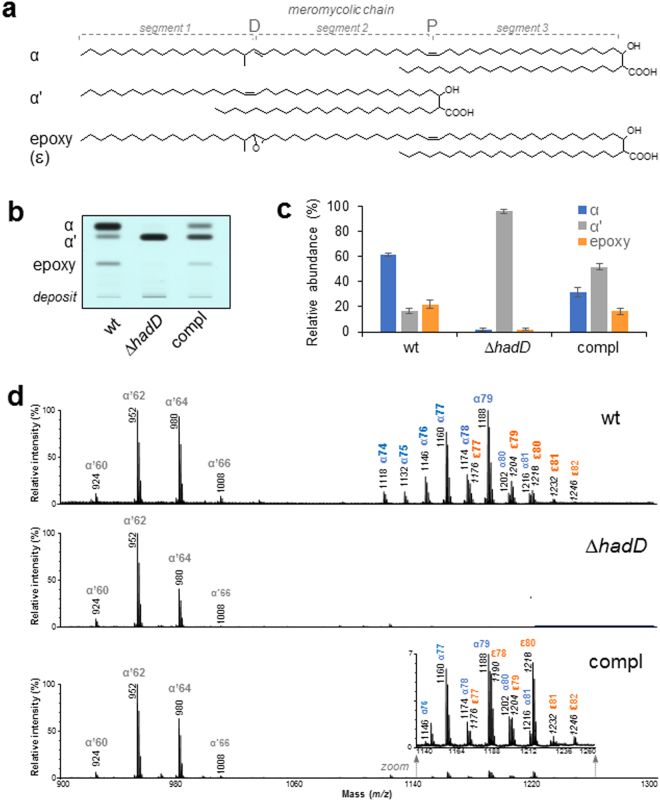
The lack of HadD profoundly changes the distribution and fine structure of the cell-wall-linked mycolic acids. (**a**) Structures of the three MA types from *M*. *smegmatis*. One of the main molecules was drawn for each type, as an example. The three segments of the meromycolic chain, delimited by the distal (D) and proximal (P) positions of the chemical functions, are indicated. (**b**) HPTLC profiles of cell-wall-linked MAMEs in the wt, ∆*hadD*, complemented (compl) and ∆*pE* strains. Five µg of each MA mixture were loaded onto a HPTLC plate developed in petroleum ether/diethyl ether (9:1, v/v), and stained by immersion in CuSO_4_ and heating. The figure is representative of four independent experiments. **(c**) MA distribution in each strain deduced from the quantification of the HPTLC band intensities as in panel (b). Data are means ± average deviations of four independent experiments. (**d**) MALDI-TOF MS spectra of the cell-wall-linked MAME mixtures. Peaks correspond to monosodium adducts. The major ion peaks are labelled with the matching MA type and the total carbon number (of the free acid form). ɛ, epoxy-MAMEs; their mass values are in italic. The spectra are representative of three independent experiments.

*M*. *smegmatis* produces three main types of mycolic acids (MAs): diunsaturated compounds called α-MAs, shorter monounsaturated compounds called α′-MAs, and monounsaturated epoxydic compounds called epoxy-MAs (Fig. 3a). A *M*. *smegmatis MSMEG_0948 (hadD)* deletion mutant was constructed (Supplementary Fig. S3) using the recombineering method to examine the impact of *hadD* gene inactivation on the biosynthesis of MAs. The total content of cell-wall linked MAs (corresponding to 93% of the MAs) remained constant after gene inactivation and represented 6.4 ± 0.4% of the delipidated bacteria dry weight. In contrast, the HPTLC MA profile changed dramatically, and the presence of α- and epoxy-mycolic acid methyl esters (MAMEs) could hardly be detected (Fig. 3b,c). This conclusion was supported by the MALDI-TOF MS analysis of the MA mixture (Fig. 3d). *M*. *smegmatis* Δ*hadD* produces almost exclusively α′-MAs, whose structures are identical to those of the wt strain, as shown by MALDI-TOF MS (Fig. 3d) and ^1^H-NMR analyses (Supplementary Fig. S4). This is the first description of such a mycobacterial strain. The partial recovery of the wt profile in the complemented strain indicates that this phenotype is directly linked to the lack of *hadD* gene. The genetic organization of the region shows that there is no possible polar effect of the deletion (Supplementary Fig. S3). Thus, the incomplete complementation may be due to the cloning of the wt *hadD* allele downstream its natural promoter, in a single copy integrative plasmid.

Consistent with the bioinformatics analyses, these data demonstrate that HadD protein plays an essential role in the biosynthesis of cell-wall linked α- and epoxy-MAs, and is dispensable for α′-MA production.

### Disruption of the glycolipid content induced by *hadD* mutation

**Figure 4:**
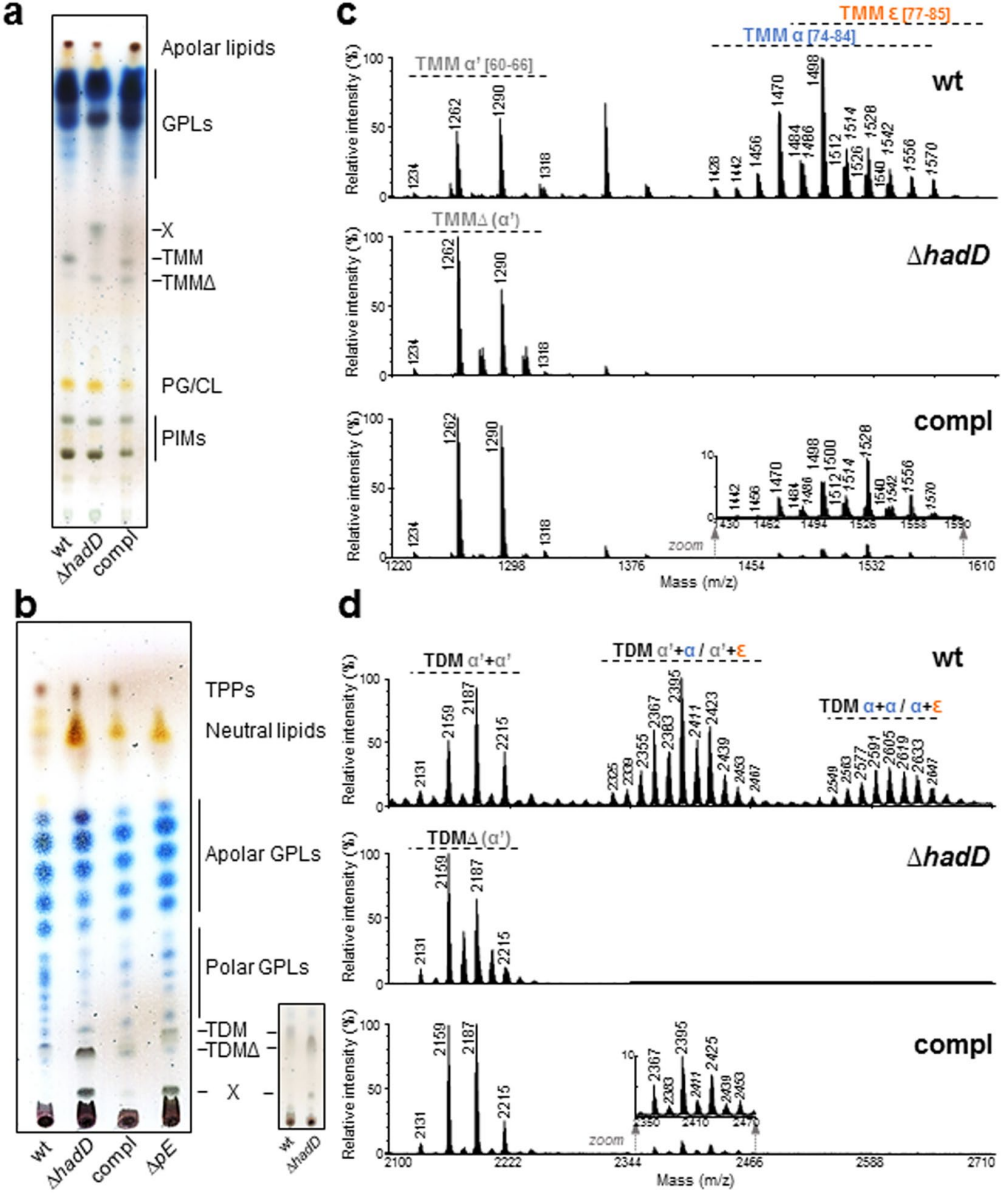
Alteration of the extractable lipid content upon *hadD* deletion. Thin layers are representative of at least three independent experiments. (**a**,**b**) TLC analyses of the total extractable lipids. Identical amounts of lipid mixtures were loaded on TLC plates developed either in CHCl_3_:CH_3_OH:H_2_O (65:25:4, v/v/v) (panel (a)) or in CHCl_3_:CH_3_OH (9:1, v/v) (panel (b)). Insert in panel (b): lower amounts of lipids were loaded to better visualize the *R*_f_ difference between TDM and TDM∆. For full picture, see Supplementary Figure S5c. The spots were revealed by anthrone spraying and heating. CL, cardiolipins; GPLs, glycopeptidolipids; PIMs, phosphatidylinositol mannosides; PG, phosphatidyl glycerol; TDM, trehalose dimycolate; TDM∆, TDMs of *M*. *smegmatis* ∆*hadD*; TMM, trehalose monomycolate; TMM∆, TMMs of *M*. *smegmatis* ∆*hadD*; TPPs, trehalose polyphleates; X, compound X. Compl, complemented strain; ∆*pE*, *M*. *smegmatis pE* deletion mutant lacking pE acyltranferase^29^. The apolar GPLs are diglycosylated and the polar GPLs are triglycosylated. (**c**,**d**) MALDI-TOF MS spectra of purified TMMs and TDMs, respectively. Peaks correspond to monosodium adducts. For clarity, *m/z* values are indicated for the major ion peaks only. Peaks of TDM α + α are present in the wt strain but globally less intense than TDM α + ɛ. The type and total carbon number of MA chains are mentioned. Mass values of epoxy-MA containing lipids are in italic. ɛ, epoxy-MA chains.

While the total amount of extractable lipids remained similar to that of the wt strain after inactivation of *hadD* (Supplementary Fig. S5a), significant qualitative changes were observed in their profile (Fig. 4a,b; Supplementary Fig. S5c). Two compounds migrating during TLC below the MA-containing lipids, *i*.*e*. the trehalose monomycolate (TMM) and the trehalose dimycolate (TDM), accumulated. These compounds, called TMMΔ and TDMΔ, were analyzed by MALDI-TOF MS (Fig. 4c,d) or MALDI-TOF/TOF MS/MS (Supplementary Fig. S6). The spectra corresponded to series of TMM and TDM homologs bearing only α′-mycoloyl chains. Consistent with this, the TLC profile showed the quasi-total absence of α- and epoxy-MAs in the extractable lipids (Supplementary Fig. S5d), as was observed for the cell-wall linked MAs (Fig. 3b,c). The MALDI-TOF mass spectra indicated that TMMΔ and TDMΔ were also present in the wt strain (Fig. 4c,d), yet as minor compounds since there were hardly or not detectable by TLC (Fig. 4a,b). Interestingly, *hadD* deletion induced a doubling of the MA content in the extractable lipids (Supplementary Fig. S5b), resulting in an increase of TDM quantity (Fig. 4b), possibly to compensate the loss of the longer chain MAs.

It is noteworthy that additional modifications of the extractable lipid profile were observed in the mutant strain. These included a marked decrease of the polar GPLs and the occurrence of a new compound (X; Fig. 4a,b). A COSY ^1^H-^1^H NMR analysis of compound X (Supplementary Fig. S7a), confirmed by a HSQC ^13^C-^1^H NMR analysis (data not shown), revealed that it is a 2,3-diacyl trehalose. MALDI-TOF MS (Supplementary Fig. S7b) and MALDI-TOF/TOF MS/MS (Supplementary Fig. S7c) analyses showed that compound X corresponds to a series of diacyl trehalose homologs previously identified as precursors of the trehalose polyphleate (TPP)^29^ and bearing a regular-size fatty acyl chain (C_14_-C_18_) and a very long polyunsaturated acyl chain (C_36:5_, C_40:6_) called a ‘phleate’^30^. In Burbaud’s survey^29^ however, these precursors accumulated as a consequence of TPP production failure in a mutant strain depleted for pE acyltranferase involved in TPP metabolism (Fig. 4b). The wt MA profile in *M*. *smegmatis* ∆*pE* (Supplementary Fig. S8) and the presence of TPP in *M*. *smegmatis* Δ*hadD* (Fig. 4b) indicated that the two biosynthesis pathways are not connected, and that the accumulation of 2,3-diacyl trehalose in the latter strain is likely a secondary effect of the disruption of the envelope lipid content.

### *HadD* inactivation affects the bacterial cell surface and envelope properties

**Figure 5:**
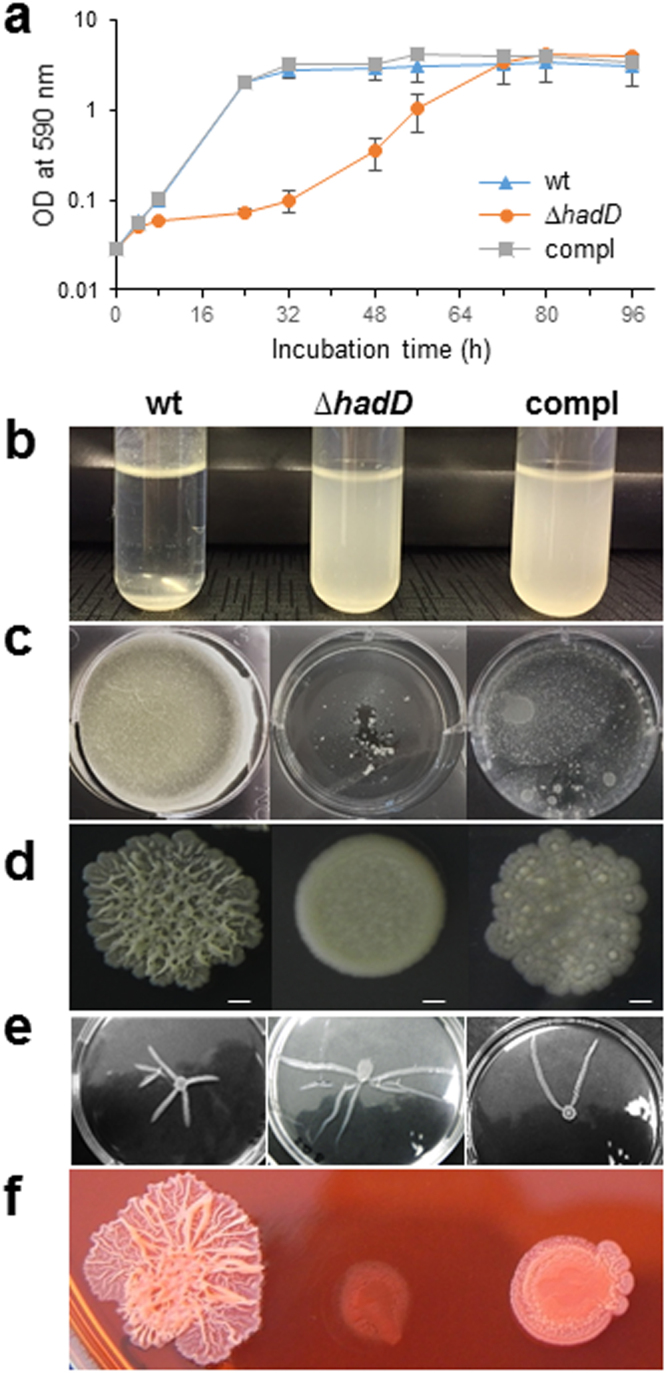
*HadD* deletion has an impact on bacterial fitness, assembly, spreading and surface hydrophobicity. Comparison of *M*. *smegmatis* wt, ∆*hadD* and complemented (compl) strains in different phenotyping assays. All the pictures are representative of three independent experiments. (**a**) Planktonic growth. Curves were established at 37 °C in 7H9-based medium supplemented with 0.05% (w/v) Tween-80. The cfu numbers determined at 0, 24 and 48 h confirmed a significant growth reduction for *M*. *smegmatis* Δ*hadD*. Data are means and standard deviations of three independent experiments. (**b**) Aggregation assays. Samples of cultures grown until saturation in 7H9-based medium without Tween were kept unshaken. Photographs correspond to the 15 min time point of the assays (Supplementary Fig. S9a). (**c**) Biofilm formation at the air-liquid interface. It was monitored on Sauton’s medium after 6 days of incubation at 37 °C. (**d**) Colony morphology. Five µl culture aliquots were spotted on 7H10-based medium. Scale bars represent 1 mm. (**e**) Sliding motility. It was monitored after 7 day incubation on semi-solid 7H9-based medium. Finger-like extensions appeared and spread outwards from the central inoculation point. *M*. *smegmatis* ∆*hadD* displayed a higher number and much longer extensions than the wt strain, whereas the complemented strain exhibited an intermediate extension number. For sliding motility quantification, see Supplementary Fig. S9b. (**f**) Colony morphology on Congo red. Five µl preculture aliquots were spotted on 7H10-based medium supplemented with 100 µg/ml Congo red. For quantification of Congo red binding, see Supplementary Fig. S9c.

The ability of mycobacteria to multiply, assemble and colonize different kinds of surfaces in the environment as well as in the host plays a determinant role on their pathogenicity. The viability of the *M*. *smegmatis* ∆*hadD* strain showed that *hadD* gene is non-essential for *M*. *smegmatis* axenic culture. Yet, the mutant bacteria displayed a reduced growth as compared to the wt strain in planktonic cultures (Fig. 5a). Moreover, they sedimented more slowly in the absence of Tween (Fig. 5b, Supplementary Fig. S9a), indicating that they aggregated to a lesser extent. On solid medium, inactivation of *hadD* gene resulted in a drastic change of the colony morphology from a rough into a smooth phenotype (Fig. 5d). The mutant bacteria had also an altered capacity to assemble into biofilms (Fig. 5c), a successful strategy developed to survive under stress and hostile conditions. In contrast, *M*. *smegmatis* ∆*hadD* strain exhibited a significantly increased sliding motility (Fig. 5e, Supplementary Fig. S9b), a flagellum-independent spreading mechanism of mycobacteria allowing their translocation on solid surfaces and probably crucial for colonization^31^. This phenomenon could be a consequence of a higher hydrophobicity of the mutant bacterial cell surface, which plays a major role in the sliding motility. Consistent with this, the *hadD*-deficient bacteria bound more efficiently the Congo red (Supplementary Fig. S9c), a hydrophobic dye that binds to lipoproteins or lipids present on the mycobacterial surface^32^. This resulted in a profound change of the colony morphology (Fig. 5f).

**Figure 6:**
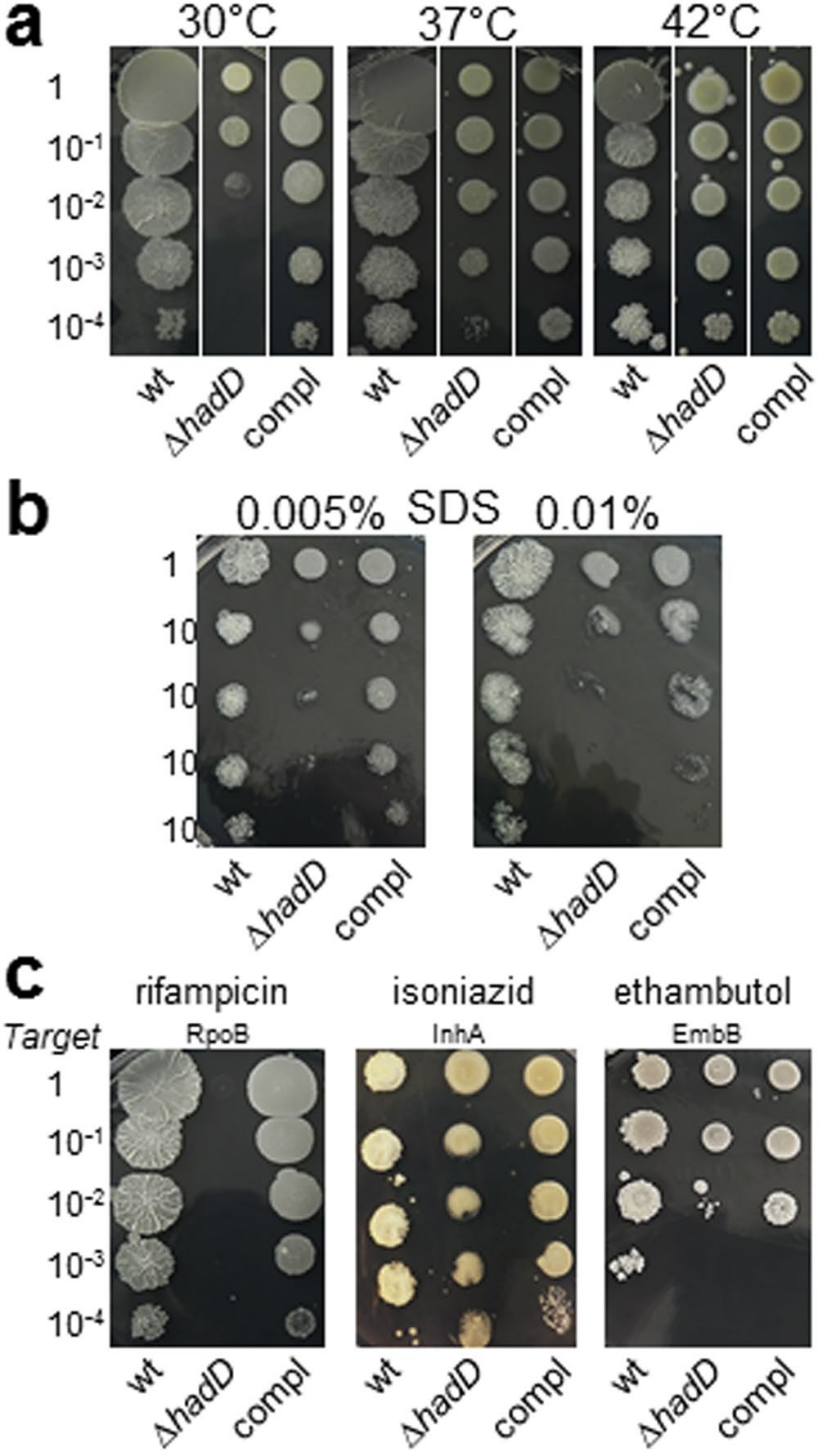
Increased sensitivity to low temperature, SDS and rifampicin upon *hadD* inactivation. Comparison of *M*. *smegmatis* wt, ∆*hadD* and complemented (compl) strains in different assays. Cultures were serially diluted then spotted on 7H10 medium supplemented with the required drug or detergent and incubated at 37 °C in standard conditions or at 30 °C and 42 °C for temperature testing. All the photographs are representative of three independent experiments. Susceptibility to (**a**) temperature, (**b**) SDS, (**c**) antimycobacterial drugs. The target protein of each drug is mentioned. RpoB is a DNA-directed RNA polymerase (*β* chain); InhA is the *trans*-2-enoyl-ACP reductase of FAS-II; EmbB is an arabinosyl transferase.

The impact of *hadD* gene inactivation on the response of mycobacteria to different stresses was then examined. The mutant strain displayed a marked sensitivity to low temperature (30 °C) and a slight sensitivity to the detergent SDS as compared to the wt strain (Table 1; Fig. 6a,b). Furthermore, it exhibited a hypersensitivity to a first line antituberculous drug, rifampicin, a large polyketide that blocks the DNA-dependent RNA synthesis. Its MIC for rifampicin was 45 fold lower than that of the wt strain (Table 1). In contrast, growth of *M*. *smegmatis* Δ*hadD* remained roughly unchanged in the presence of two other firstline TB drugs, isoniazid and ethambutol, which inhibit the biosyntheses of cell wall mycolic acids and polysaccharides, respectively (Table 1; Fig. 6c).

**Table 1:** Impact of *hadD* deletion on the sensitivity of *M*. *smegmatis* to antibiotics and SDS in planktonic cultures.

*M*. *smegmatis* strain	Minimal inhibitory concentration^*a*^			
	Rifampicin (µg/ml)	Isoniazid (µg/ml)	Ethambutol (µg/ml)	SDS (%)
wt	16.9 ± 4.8	7.1 ± 0.9	3.9 ± 1.2	0.016 ± 0.000
Δ*hadD*	0.37 ± 0.13	7.7 ± 0.2	3.1 ± 0.0	0.007 ± 0.001
complemented	3.1 ± 0.9	6.4 ± 1.6	3.1 ± 0.0	0.012 ± 0.004

^a^MIC values (means ± average deviations) measured by MTT assays were determined as the lowest concentration of compound yielding 99.9% inhibition of the bacterial growth.

These data altogether reveal an upheaval of both the bacterial cell surface and the envelope properties of *M*. *smegmatis* upon *hadD* inactivation, with consequences on the envelope integrity and permeability as well as the organization and motility of the bacterial populations. The complementation of the mutant strain by a wt *hadD* copy generally resulted in intermediate phenotypes (Figs 5 and 6; Supplementary Fig. S9; Table 1), showing that these phenomena are directly linked to *hadD* deficiency.

## Discussion

A combination of eGFP-based pull-down, proteomics and bioinformatics analyses led us to discover a novel partner protein of the mycobacterial FAS-II system, MSMEG_0948 or HadD, in the NTM *M*. *smegmatis*. HadD is the closest homolog of HadA and HadC proteins, of which it most likely adopts the single hot dog fold. HadA and HadC are the substrate-binding subunits of HadAB and HadBC dehydratases of the FAS-II system, while HadB represents their catalytic subunit^21,22^. Interestingly, HadD holds a well conserved catalytic dyad within a degenerate version of the catalytic hydratase 2 motif found in HadB and absent from HadA and HadC, strongly suggesting it is a (*R*)-specific hydratase/dehydratase. Consistent with this, activity assays performed with purified HadD showed that this protein possesses a hydratase/dehydratase function. Most importantly, HadD is ubiquitous among mycobacteria but, unlike most FAS-II enzymes and particularly HadAB dehydratase, has no ortholog in the other mycolic acid-producing genera. Thus, this protein appears specific to the mycobacterial cells, including *M*. *leprae* genome that retains a minimal set of genes for cell-wall biosynthesis^33^. This suggests that its function is not linked to the construction of the common meromycolic medium chain backbone realized by most *Corynebacteriales* FAS-II systems but is rather dedicated to a reaction step specifically required for biosynthesis of the mycobacterial MAs. This is also the case of HadC protein that, associated to HadB, is involved in the late FAS-II elongation steps generating the longest meromycolic chains in mycobacteria^14^. Yet, the MA profile observed for *M*. *smegmatis hadD* mutant is completely different from that of *M*. *smegmatis hadC* mutant that still produces the three MA types, namely α, α′ and epoxy, although in proportions different from that found in the wt strain^23^. Furthermore, several physiological parameters, such as the planktonic growth rate, sliding motility, surface hydrophobicity and sensitivity to temperature and SDS are differently affected in both mutants^23^. These data altogether clearly show that HadD function is distinct from that of HadC in *M*. *smegmatis*.

**Figure 7:**
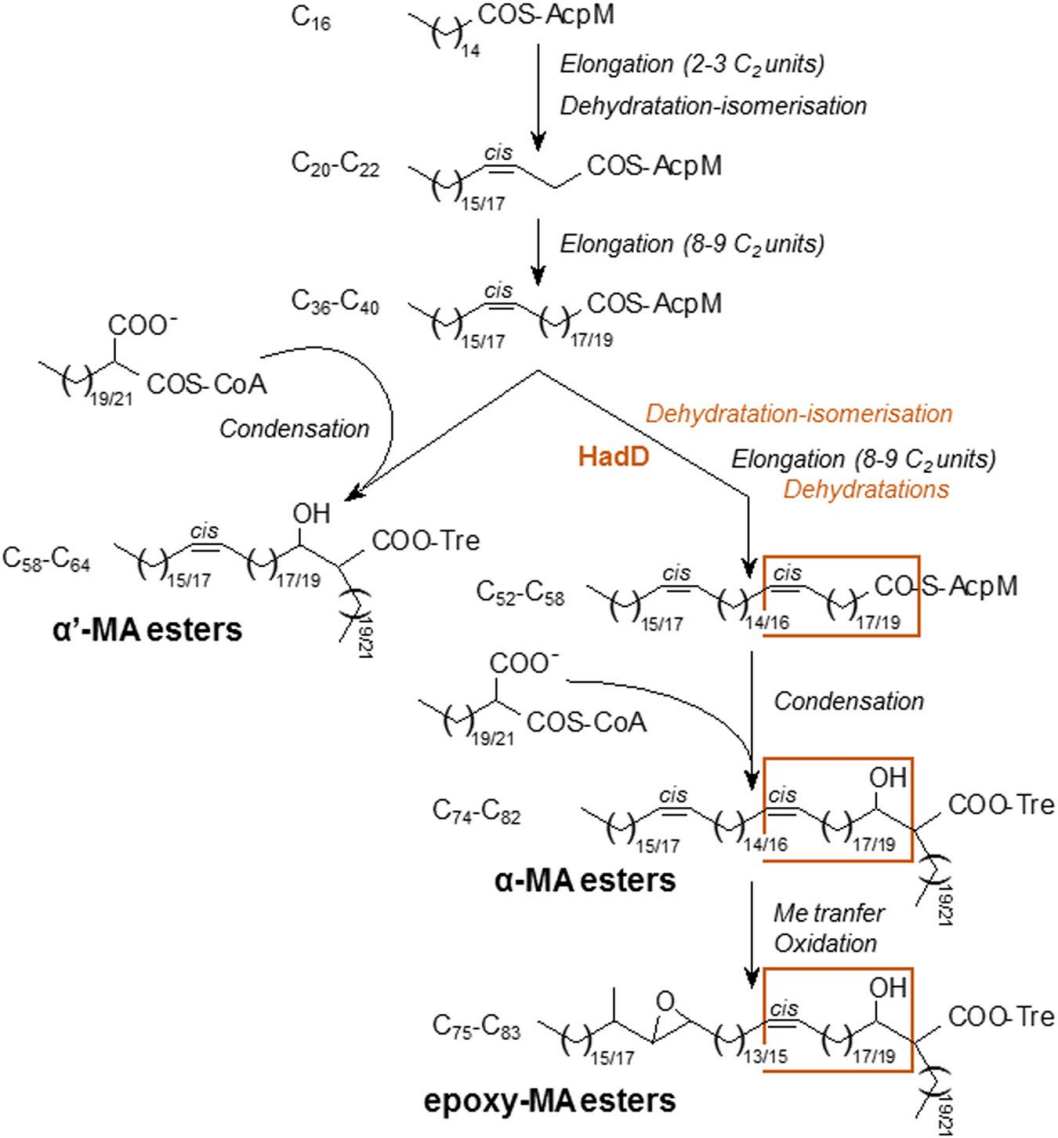
Proposed function of HadD in the mycolic acid biosynthesis pathway in *M*. *smegmatis*. The biosynthesis process of the meromycolic chain starts from the methyl-terminal end. After a first set of FAS-II elongation cycles, the possible dehydratation-isomerization of a C_20_-C_22_ 3-hydroxyacyl-AcpM intermediate would allow the introduction of the future distal *cis* double bond (Fig. 3a) in the meromycolic chains. After a second set of FAS-II elongation cycles, a Claisen-type condensation of the resulting short α′-meromycoloyl chains with a carboxy-C_22_/C_24_ fatty acyl-CoA by Pks13 enzyme, followed by transfer onto trehalose (Tre) and reduction, generates the trehalose α′-MA esters. A second possible dehydratation-isomerization of the α′-meromycoloyl chains would introduce the future proximal *cis* double bond (Fig. 3a). After a third set of FAS-II elongation cycles including (3*R*)-hydroxyacyl-AcpM dehydratation steps, the condensation of the resulting long α-meromycoloyl chains with a carboxy-C_22_/C_24_ fatty acyl-CoA generates the trehalose α-MA esters. Finally, methyl transfer and oxidation reactions allow the biosynthesis of the epoxy-MA esters. HadD would catalyze either the dehydratation-isomerization or the dehydratation reactions during the synthesis of the third meromycolic segment (orange frame) (see also Fig. 3a). The total carbon number of the different acyl chains are mentioned.

The inactivation of *hadD* in *M*. *smegmatis* abolishes the production of both α- and epoxy-MAs. This phenomenon as well as common structural features between these two MA types and the chronological sequence of their formation in wt bacteria^34^ strongly support the existence of a direct biosynthetic filiation between these two MA types. Thus, HadD function is dedicated to the synthesis of these long chain MAs and would be required for the formation of the third hydrocarbon segment present in the epoxy- and α-MAs and absent in the α′-MAs (Figs 3a, 7). Given that HadD belongs to the (*R*)-specific hydratases/dehydratases family, it might be responsible either for the introduction, using a dehydratation-isomerization mechanism, of the second (proximal) *cis*-double bond into the meromycolic chain, or for the (3*R*)-hydroxyacyl dehydration step of the FAS-II elongation cycles devoted to the third meromycolic segment (Fig. 7). Therefore, blocking such steps linked to the α/epoxy-MA biosynthetic split would completely stop their production. Unless knowing their atomic structure, it is impossible to distinguish simple dehydratases from dehydratase-isomerases, which display similar global 3D structures and catalytic machineries, and exhibit subtle differences in the shape of the substrate-binding tunnel^35^. The dehydratase-isomerase family uses an anaerobic catalytic mechanism^36^ distinct from the molecular oxygen-dependent mechanism used by the desaturases^37^ such as *M*. *smegmatis* DesA1 desaturase that may be involved in the introduction of one double bond in the α-MAs, as previously proposed^20^. The opportunistic pathogen *Pseudomonas aeruginosa* possesses two distinct pathways for unsaturated fatty acid biosynthesis^38^. Like in *Escherichia coli*, the main one uses an anaerobic mechanism dependent on the dehydratase-isomerase FabA belonging to its FAS-II system. The second one is an aerobic inducible pathway governed by the desaturases DesA and DesB^38^. Thus, it is plausible that introduction of double bonds into MAs is also governed by two different mechanisms that would be required to adapt to changes of environmental conditions and especially of oxygen levels.

Profound physiological changes were observed in the *hadD* deficient mutant, where the major component of the mycomembrane corresponds to the medium-size α′-MA alone instead of a combination of the three MA types. To our knowledge, this is the first description of a mycobacterial strain holding only α′-MA. The NTM *M*. *smegmatis* can survive *in vitro* with this sole MA. Yet, its planktonic growth is considerably slowed down. Furthermore, its capacities to aggregate or to assemble into colonies or biofilms and to spread by sliding motility are altered, likely due to an increased hydrophobicity of its cell surface reflecting an envelope architecture upheaval, but also to a change in MA content, which plays an important role in biofilm growth^12^. Interestingly, inactivation of *hadD* also affects, probably indirectly, the metabolism of two families of glycolipids, the TPPs and GPLs, whose biosyntheses are orchestrated by two adjacent clusters on *M*. *smegmatis* chromosome^29^, located very far (≥536 kb) from *hadD*. There is a close correlation between the mutant strain features and the marked reduction observed in the content of polar (triglycosylated) GPLs, which are surface-exposed lipids, therefore playing an important role in the cell surface properties, and condition the sliding motility, aggregation and Congo red absorption of *M*. *smegmatis*^39^. Moreover, the modification of the envelope composition in MA-containing compounds in the mutant affects the integrity of the bacterial envelope, causing an increased sensitivity to low temperature, SDS and particularly to rifampicin antibiotic. Reported data suggested that the cell wall fluidity is directly correlated to the MA length^10^. One would expect that the *hadD*-deficient mutant, holding solely medium-size α′-MAs, would have a more fluid mycomembrane favorable to rifampicin diffusion and low temperature tolerance. Nonetheless, subtle structural features such as the decorations on the meromycolic chains, have proved to have also a great impact on the folding and packing of the MA chains within the mycomembrane^40^ and consequently on cell wall permeability^10^.

The emergence of multidrug-resistant *M*. *tuberculosis* strains severely compromises TB treatment^1^. Furthermore, the intrinsic resistance of NTM to most conventional antibiotherapies and long-term treatment regimens of moderate efficacy are two main factors of the mortality observed for NTM infection cases^5^. Thus, there is an urgent need for novel therapeutic strategies for the treatment of TB and NTM diseases^1,5^. The loss of capacities of the mycobacterial *hadD* mutant to multiply, assemble and colonize different kinds of surfaces as well as its hypersensitivity to the firstline anti-mycobacterial drug rifampicin makes HadD protein a very attractive target for future drug development. Using a HadD inhibitor in drug combinations could also represent a relevant approach to improve the antibiotherapy against pathogenic mycobacteria.

## Methods

### Pull-downs

For pull-down experiments using HadAB enzyme as a bait, a *M*. *smegmatis* strain deleted for the endogenous *hadABC* operon (*MSMEG_1340-1341-1342*)^23^ was complemented by the plasmid pKW08::*egfp-hadABC*. The latter was constructed by cloning the *hadABC* operon of *M*. *tuberculosis* H37Rv (*Rv0635-0636-0637*) in the tetracycline-inducible vector pKW08::*egfp* (kindly provided by A. Dziembowski, University of Warsaw, Poland)^41,42^ between BamH1 and HindIII sites, downstream of the enhanced Green Fluorescent Protein (eGFP) tag followed by the Tobacco Etch Virus (TEV) cleavage site. The control strain used for these experiments was *M*. *smegmatis* ∆*hadABC*/pKW08::*egfp* complemented by the pGBT::*hadABC* vector^23^. The resulting strains were grown in Middlebrook 7H9 broth (Difco) supplemented with 50 µg/ml hygromycin, 10% (w, v) ADC and 0.2% (v/v) glycerol, plus 37.5 µg/ml kanamycin for the control strain, and the production of the protein(s) was increased by adding 50 ng/ml tetracycline (Sigma) during the exponential phase. The affinity purification method used was adapted from a published protocol^42^. Bacteria were centrifuged at 10,000 g for 30 min at 4 °C, resuspended in lysis buffer: 50 mM potassium phosphate buffer pH 7.6, 100 mM NaCl, 1 mM DTT, 2 mM AEBSF (Euromedex), 1% (v, v) Triton X-100, 5 µg/ml DNAse I and 5 µg/ml RNase A, and lysed using a Biorupteur Pico (Diagenode) for 20 cycles (30 sec on, 30 sec off). The lysate was clarified by centrifugation at 20,000 g for 20 min at 4 °C and incubated in the presence of GFP-Trap®_MA magnetic beads (Chromotek) for 3 h at 4 °C. After four washes with 10 mM Tris buffer pH 7.6, 150 mM NaCl, 0.1% (v,v) Triton X-100, proteins were eluted by cleavage using 10 units AcTEV™ protease (Fisher) in 10 mM Tris buffer pH 7.6, 150 mM NaCl, 0.5 mM EDTA, 1 mM DTT, overnight at 4 °C.

### Proteomics analyses

Sample preparation and nanoLC-MS/MS for proteomic analysis were performed as previously described^43^. Briefly, after reduction and alkylation of cysteine residues, eluted proteins were concentrated in one band by 12% SDS-PAGE. The proteins were then digested with trypsin (1 µg trypsin/50 µg proteins) overnight at 37 °C. The resulting peptides were extracted from the gel, then dried and finally resuspended in 2% acetonitrile, 0.05% TFA. Peptides were analyzed by nanoLC-MS/MS using an UltiMate 3000 RSLCnano system (Dionex, Amsterdam, The Netherlands; 5 to 50% gradient of 80% acetonitrile, 0.2% formic acid for 60 min at 300 nl/min flow rate) coupled to a Q-ExactivePlus mass spectrometer (ThermoScientific, Bremen, Germany). Raw MS files were analyzed by MaxQuant version 1.5.5.1. Data were searched with the Andromeda search engine against *Mycobacterium smegmatis* entries of the Swissprot-Trembl protein database (UniProtKB/Swiss-Prot Knowledgebase release 2017_03, *M*. *smegmatis* MC2 taxonomy, 8805 entries) and a list of potential contaminant sequences provided in MaxQuant1.5.5.1. Bioinformatics data analysis was performed as described previously^43^. The dataset contains mass spectrometry results from the analysis of four biological replicates. The statistical proteomics analysis was performed using the Perseus 1.5.3.0 software on the LFQ intensities from the “proteinGroups” table of MaxQuant.

### Sequence analyses and homology modeling

Analyses of genome sequences were done via the SmegmalList (http://mycobrowser.epfl.ch/smegmalist.html)^24^ and the NCBI (www.ncbi.nlm.nih.gov/genomes/lproks.cgi) web sites. Sequence alignments were performed using BLAST and Clustal Omega softwares^44,28^, with default parameters. In particular, the presence of HadD in mycobacteria and other genera of the *Corynebacteriales* order, i.e. *Corynebacterium*, *Nocardia*, *Rhodococcus*, *Gordonia*, *Tsukamurella*, *Segniliparus*, *Williamsia*, *Dietzia and Tomitella*, was analyzed by BlastP searches^28^ against fully sequenced genomes available at NCBI web site, using MSMEG_0948 protein sequence (Supplementary Figure S1a; database accession number: A0QR13**)** as a probe. Structure prediction, homology modeling and model evaluation were performed through the @TOME 2.3 Platform^45^. Briefly, the structural alignment research against PDB was done using HHsearch. The 3D model was built using the crystal structure of *M*. *tuberculosis* HadA protein (4RLJ)^46^ (41% sequence identity with MSMEG_0948) as a template in the program MODELLER V9^47^. The model was evaluated through the internal Modeller energy score and Modeller, QMean, Verify3D, Dope, Dfire and Errat softwares. The good scores obtained (e.g. Modeller: 604.39; QMean: 0.51) indicated the good quality of the model.

### Protein production and purification

The *MSMEG_0948* gene from *M*. *smegmatis* mc²155 was amplified by PCR and the product was ligated into pET-15b vector (Novagen) between *Nde*I and *Bam*HI restriction sites; the absence of mutation was verified by sequencing. For expression of the N-terminal His_6_-tagged HadD protein, *E*. *coli* Rosetta^TM^ (DE3) strain (Novagen) was transformed by pET-15b:: *MSMEG_0948* construct and grown at 37 °C under shaking (200 rpm) in Terrific broth medium (Euromedex) supplemented with carbenicillin (50 µg/ml) and chloramphenicol (30 µg/ml). Cultures were stopped at an OD_600_ of 0.8 and left at 13 °C for 30 min. Gene expression was induced by addition of 1 mM IPTG, and bacteria were grown further for 24 h at 13 °C and 200 rpm. After centrifugation, the cell pellet was resuspended in 50 mM HEPES buffer pH 7.5 containing 500 mM NaCl, 0.5% Triton X-100, 10 mM imidazole, 1 mM AEBSF and 0.5 mg/ml lysozyme. After freezing at −80 °C and thawing, 5 µg/ml DNase I, 10 µg/ml RNase A and 10 mM MgCl_2_ were added to the bacterial lysate. After centrifugation at 27,000 *g* for 20 min at 4 °C, the supernatant was loaded onto TALON® superflow metal affinity resin (Clontech) equilibrated with 50 mM HEPES buffer pH 7.5, 300 mM NaCl and 10 mM imidazole. After extensive washes at 10 mM then 30 mM imidazole in the same buffer, the H-HadD protein was finally eluted using 150 mM imidazole (Supplementary Fig. S2). Imidazole was then removed by dilution of the elution fractions in 50 mM HEPES pH 7.5, 300 mM NaCl buffer then concentration by ultrafiltration, and the protein solution was stored at −20 °C after addition of 50% (v/v) glycerol.

### Enzyme activity assays

Hydratase activity was monitored at 263 nm using a UVmc2 spectrophotometer (SAFAS) in the presence of *trans*-2-dodecenoyl-CoA (C_12:1_-CoA; ∆A of 0.67 for a variation of concentration of 100 µM) synthesized as previously described^48^. Kinetic assays were performed in a quartz cuvette for 40 sec at room temperature, in 100 mM sodium phosphate buffer pH 7.0 in the presence of 10 µM C_12:1_-CoA. After equilibration of the baseline, reactions were started by adding purified 50–200 nM H-HadD protein. Control experiments lacking the protein were realized.

### Construction of the deletion mutant and the complemented strain, and culture conditions

The *M*. *smegmatis* ∆*hadD* (*MSMEG_0948*) mutant was constructed from *M*. *smegmatis* mc² 155 strain by the recombineering system^49^ with some method modifications as described previously^50^. Briefly, an allelic exchange segment (AES) was generated with a two-step PCR procedure. First, three PCRs were performed to amplify two ~500-bp fragments corresponding to the upstream and downstream regions of *MSMEG_0948* ORF and the 1352-bp streptomycin resistance cassette (*aadA*^+^ gene). Second, the three PCR products were used for a three-fragment fusion PCR to generate the AES. The product was used to transform a competent recombineering strain. Selection was performed on 7H10 medium containing streptomycin (20 µg/ml). This resulted in the replacement of the target gene by the streptomycin resistance cassette, as shown by PCR analysis (Supplementary Fig. S3) and sequencing. The recombineering plasmid (pJV53) carrying a hygromycin resistance cassette was cured from the mutant by successive cultures without selection, and its loss was checked by plating on hygromycin-containing solid medium. For complementation, a wt copy of *MSMEG_0948* gene was introduced between the *Not*I and *Eco*RI restriction sites of the integrative plasmid pUC-Gm-Int^51^ (kindly provided by T. Parish, Seattle) and the resulting plasmid was used to transform *M*. *smegmatis* ∆*MSMEG_0948* strain. Both the parent *M*. *smegmatis* mc²155 and *M*. *smegmatis* ∆*MSMEG_0948* strains were also transformed by the empty pUC-Gm-Int plasmid. The standard culture conditions were at 37 °C either under shaking (190 rpm) in Middlebrook 7H9 broth (Difco) supplemented with 0.2% glycerol and gentamycin (5 µg/ml), or on plates of Middlebrook 7H10 medium (Difco) supplemented with 0.2% glycerol and gentamycin (10 µg/ml). *M*. *smegmatis* ∆*pE* mutant, where the *pE* (*MSMEG_0412*) gene coding for pE acyltranferase has been completely deleted and replaced by a kanamycin resistance cassette^29^, was kindly provided by C. Chalut (IPBS-CNRS, Toulouse). The culture medium used for this strain was the Middlebrook 7H9 broth supplemented with 0.2% glycerol and kanamycin (50 µg/ml).

### Planktonic and biofilm growths, and colony morphology

Growth curves were realized by measuring the OD at 600 nm of liquid cultures incubated at 37 °C under shaking (190 rpm) in Middlebrook 7H9 broth (Difco) supplemented by 0.2% glycerol, 0.05% (w/v) Tween-80, 10% ADC (Difco) and 5 µg/ml gentamycin. The cfu numbers were counted at time points 0, 24 and 48 h after spreading serial dilutions on agar plates to verify the growth discrepancy of *M*. *smegmatis* Δ*hadD* strain. For biofilm formation^52^, exponential precultures were grown in the above medium then adjusted at OD_600_ ~ 1. Fifty µl of each strain were deposited at the surface of 5 ml Sauton’s medium containing gentamycin (5 µg/ml) in 6-well plates, and incubated at 37 °C for 6 days. For observation of colony morphology, liquid precultures were done in 7H9-based broth containing 0.05% (w/v) Tween-80 until saturation, then adjusted to the same OD. Five µl aliquots were spotted on Middlebrook 7H10 medium (Difco) containing 0.2% glycerol and incubated at 37 °C for 3 days.

### Aggregation assays

For the aggregation assays based on a published protocol^53^, the *M*. *smegmatis* strains were grown in Middlebrook 7H9 broth supplemented with 0.2% glycerol and gentamycin (5 µg/ml), without Tween, at 37 °C under shaking (190 rpm) until saturation. Five ml culture aliquots were transferred into tubes (10 × 70 mm) then homogenized and the OD_600_ measured at time 0 min (OD_T0_). Tubes were kept unshaken for 15 min at room temperature and the OD_600_ were measured at 5 min intervals (OD_T_). The aggregation indexes (AI) were calculated using the following equation: AI = 100 × (OD_T0_ − OD_T_)/OD_T0_.

### Congo red binding assays

The Congo red binding assay^54^ was adapted as follows. Mycobacteria were cultivated for 3 days at 37 °C under shaking (250 rpm) in Middlebrook 7H9 broth (Difco) supplemented by 0.2% glycerol, 0.05% (w/v) Tween-80, 5 μg/ml gentamycin and 100 µg/ml Congo red. After centrifugation at 3000 g for 10 min, the bacterial pellets were resuspended in 1 ml water and sonicated for 15 min in a water bath. Bacteria were then washed extensively with water until the supernatant was colorless. Bacteria were resuspended in 1 ml of acetone, vortexed and allowed to sit at room temperature for 1 h. Cells were then pelleted by centrifugation and the Congo red in the supernatants was measured spectrophotometrically at 488 nm. The Congo red binding index was determined as the ratio: OD_488_/dry weight (mg) of the cell pellet. For observation of colony morphology in the presence of Congo red, liquid precultures were done in 7H9-based broth containing 0.05% (w/v) Tween-80 until saturation, then adjusted to the same OD. Five µl aliquots were spotted on Middlebrook 7H10 medium (Difco) containing both 0.2% glycerol and 100 µg/ml Congo red (Sigma) and incubated at 37 °C for 3 days.

### Sliding motility assays

The sliding motility assays were performed as described^31^ with slight modifications. Briefly, the different *M*. *smegmatis* strains were first grown few days at 37 °C on M63 (Amresco) agar plates supplemented with 1 mM MgCl_2_, 0.2% glycerol and gentamycin (10 µg/ml). Plates of M63 medium solidified with 0.3% agar with no added carbon source were inoculated in their center from single colonies by poking with a sterile toothpick. The motility was evaluated visually after incubation at 37 °C for 5 days. The sliding motility index was defined as the product: radius of the spreading surface (cm) × number of finger-like spreading extensions. For photographs, liquid precultures were done in 7H9-based broth containing 0.05% (w/v) Tween-80 and adjusted to the same OD. Ten µl aliquots were spotted onto semi-solid Middlebrook 7H9 broth containing 0.3% agar without additional carbon source and incubated at 37 °C for a week.

### Drug, detergent and temperature sensitivity testing

The minimum inhibitory concentrations (MIC) for several antibiotics and SDS were determined in 7H9-based medium (without tween) by using a colorimetric microassay based on the reduction of 3-(4,5-dimethylthiazol-2-yl)-2,5-diphenyltetrazolium bromide (MTT, Sigma-Aldrich) into formazan by metabolically active cells, as described^55^. For assays on solid medium, liquid precultures were done in 7H9-based broth containing 0.05% (w/v) Tween-80, adjusted to the same OD and serially diluted. Five µl aliquots of each dilution were spotted onto Middlebrook 7H10 medium. When required, the tested drug or detergent was added to the medium: rifampicin (2 µg/ml), isoniazid (1 µg/ml), ethambutol (1 µg/ml) or SDS (0.005 or 0.01%). Cultures were incubated for 3 days at 37 °C (for drug/detergent testing) as well as 30 °C and 42 °C (for temperature testing).

### Lipid extractions and (HP)TLC analyses

The total extractable lipids were extracted from wet bacterial pellets by three successive extractions using distinct mixtures of CHCl_3_:CH_3_OH (1:2, 1:1 and 2:1, v/v). The fractions were pooled, washed with water, and dried. For TLC analysis, equivalent weights of total lipid fraction from each strain were spotted on silica gel 60 plates (Merck), which were developed either in CHCl_3_:CH_3_OH (9:1, v/v) or in CHCl_3_:CH_3_OH:H_2_O (65:25:4, v/v/v), as indicated. Compounds were revealed by spraying with 0.2% (w/v) anthrone in concentrated H_2_SO_4_ followed by heating at 100 °C.

MAs were extracted both from the delipidated bacterial residues and the total extractable lipids after saponification as previously described^56^, dried and weighted. The dry weights of MAs were normalized by the dry weights of the bacterial residues. MAs were methylated with diazomethane. The relative abundance of the different MAME classes from each strain was determined by loading a fixed amount (5 μg) of MA mixture onto a HPTLC silica gel 60 plate (Merck) with a Camag ATS4 apparatus. The plate was developed in petroleum ether/diethyl ether (9:1, v/v) using a Camag ADC2 device and stained by immersion in 10% (w/v) CuSO_4_ (in H_3_PO_4_:CH_3_OH:H_2_O, 8:5:87, v/v/v) with a Camag CID3 apparatus, followed by heating at 150 °C for 20 min. The MAMEs were quantified by absorption measurement at 400 nm with a Camag Scanner 3 device using Wincats software. For TLC analyses of MAMEs, equivalent weights of compounds were spotted on silica gel 60 plates (Merck), which were developed in dichloromethane and revealed by spraying either by CuSO_4_ solution (see above).

### Purification of TMM/TDM, and compound X

TMM, TDM and compound X were separated from other lipids of the total extractable lipids by anion-exchange chromatography with a quaternary methylammonium column (Pall) using increasing percentages (0 to 100%) of CH_3_OH in CHCl_3_, and analyzed by TLC. The purification was achieved by preparative TLC using silica gel 60 plates (Merck) developed in CHCl_3_:CH_3_OH:H_2_O (65:25:4, v/v/v). The compounds of interest were then scraped off and extracted from silica gel three times with CHCl_3_/CH_3_OH (8:2, v/v).

### MALDI-TOF MS and NMR spectroscopy

Matrix assisted laser desorption ionization-time of flight mass spectrometry (MALDI-TOF MS) and MS/MS analyses were performed in the positive ionization and reflectron mode, using the 5800 MALDI-TOF/TOF Analyzer (Applied Biosystems/ABsciex) equipped with a Nd:YAG laser (349 nm wavelength). MS and MS/MS spectra were acquired with a total of 2,500 shots at a fixed laser intensity of 4,000 (instrument-specific units) and 400-Hz pulse rate for MS, and a total of 5000 shots at a fixed laser intensity of 6000 (instrument-specific units) and 1000-Hz pulse rate for MS/MS. For MS/MS data acquisition, the fragmentation of selected precursors ions was performed at a collision energy of 1 kV using air as collision gaz. Reaction media of enzyme activity assays were first diluted 10 folds in water. 1 μl samples were spotted onto the target plate, mixed with 1 μl of matrix [10 mg/ml of 2,5-dihydroxybenzoic acid (Sigma-Aldrich) in water:acetonitrile, 8:2 (v/v)]. Lipid samples were dissolved in chloroform and were directly spotted onto the target plate as 0.5 µl droplets, followed by the addition of 0.5 µl of matrix solution [10 mg/ml of 2,5-dihydroxybenzoic acid in CHCl_3_/CH_3_OH (1:1, v/v)]. Samples were allowed to crystallize at room temperature. MS data were acquired using the instrument default calibration.

1D and 2D ^1^H-NMR, ^1^H-^1^H-COSY, ^1^H-^1^H-TOCSY (100 ms), ^1^H-^13^C-HSQC and ^1^H-^13^C-HMBC experiments were recorded at 298° K using a 600-MHz Bruker Avance III spectrometer (Bruker Biospin) equipped with a TCI cryoprobe. Purified compound X was dissolved in CDCl_3_/CD_3_OD (8:2, v/v). Chemical shifts were referenced to CDCl_3_ (δH 7.26 ppm and δC 77 ppm).

## Electronic supplementary material

Supplementary Figures
