# Supplementary material for: HadD, a novel fatty acid synthase type II protein, is essential for alpha- and epoxy-mycolic acid biosynthesis and mycobacterial fitness
Source: Sci Rep. 2018 Apr 16;8:6034. doi: 10.1038/s41598-018-24380-5 (PMC5902629; doi:10.1038/s41598-018-24380-5)
Supplement: Supplementary file 1 — Supplementary Figures [file 41598_2018_24380_MOESM1_ESM.pdf]

HadD, a novel fatty acid synthase type II protein, is essential for alpha- and epoxy-mycolic acid biosynthesis and mycobacterial fitness.

Cyril Lefebvre[1], Richard Boulon[1], Manuelle Ducoux[2], Sabine Gavalda[1], Françoise Laval[1], Stevie Jamet[1], Nathalie Eynard[1], Anne Lemassu[1], Kaymeuang Cam[1], Marie-Pierre Bousquet[2], Fabienne Bardou[1], Odile Burlet-Schiltz[2], Mamadou Daffé[1], Annaïk Quémard[1*].

Affiliations:
[1]Département Tuberculose & Biologie des Infections, [2]Département Biologie Structurale & Biophysique, Institut de Pharmacologie et de Biologie Structurale, UMR5089, Université de Toulouse, CNRS, UPS, 31077 Toulouse Cedex 04, France.

**Correspondence and requests for materials should be addressed to A.Q. (email: annaik.quemard@ipbs.fr).*

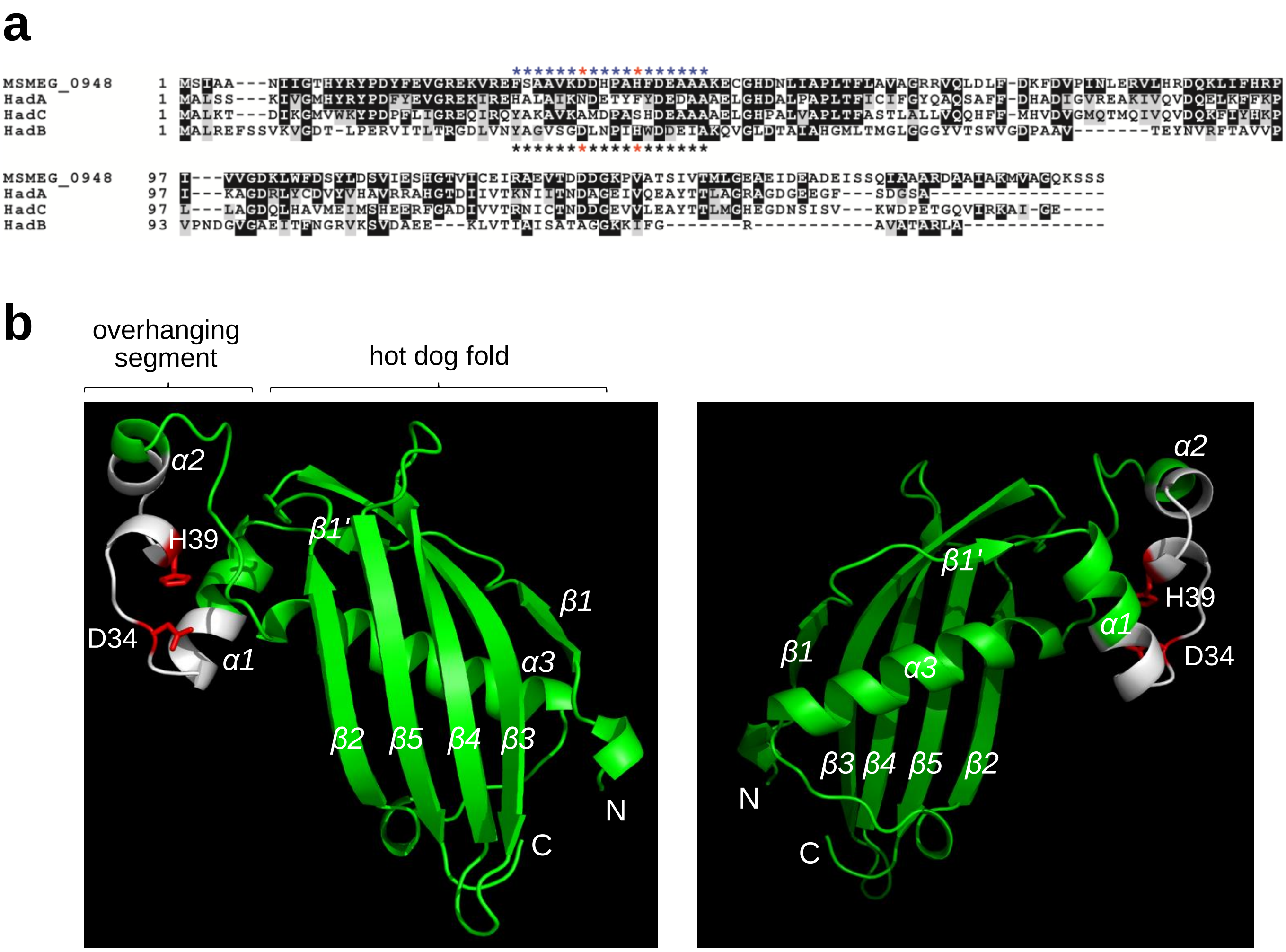

Supplementary Figure S1. MSMEG_0948 (HadD) protein would be a hot dog folded (*R*)-specific hydratase/dehydratase. (**a**) Sequence alignment of MSMEG_0948 (HadD) with HadA, HadB and HadC proteins from *M. smegmatis*. Black and gray shadings indicate strictly conserved and similar residues, respectively. MSMEG_0948 shares a sequence identity of 41% with HadA, 35% with HadC, and only 21% with HadB (Clustal Omega scores). The HadA/HadC and HadA/HadB identity rates are 46% and 19%, respectively. MSMEG_0948 bears a degenerate hydratase 2 motif 'F-x(2)-a-x(2)-**D**-x(2)-P-a-**H**-x(5)-A' (uppercase: strictly conserved; lowercase: similar residue) indicated by blue stars; the putative catalytic Asp and His residues are labeled by red stars. The hydratase 2 motif '[YF]-x(1,2)-[LIVG]-[STGC]-G-D-x-N-P-[LIV]-H-x(5)-[AS]' of HadB is indicated by black stars. Alignment was performed by using Clustal Omega program, and the figure was shaped with Boxshade. Database accession numbers: MSMEG_0948, A0QR13 (177 aa); HadA, A0QS40 (158 aa); HadB, A0QS41 (142 aa); HadC, A0QS42 (169 aa). (**b**) Homologous modelling. Ribbon representation of MSMEG_0948 monomeric model. MSMEG_0948 would organize, like HadA and HadC, as a single 'hot dog' fold made of a central α-helix wrapped into a five-stranded antiparallel β-sheet. The degenerate hydratase 2 motif (residues 28-45) is colored in white, except the catalytic residues (Asp34 and His39) which are in red. The secondary structure elements are labelled according to HadA structure[1]. Figure performed using PyMol software (http://www.pymol.org).

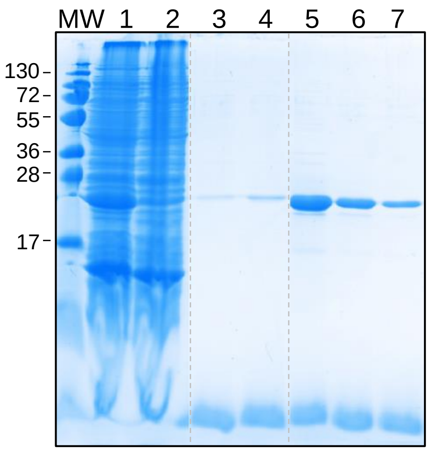

Supplementary Figure S2. Purification of HadD. Coomassie blue-stained SDS-PAGE of the purification steps on TALON® superflow metal affinity resin. MW, molecular weight markers; 1, total soluble proteins of cleared bacterial lysate; 2, flow through; 3, wash fraction at 10 mM imidazole; 4, wash fraction at 30 mM imidazole; 5-7, elution fractions at 150 mM imidazole. H-HadD theoretical molecular mass: 21,7 kDa. The dividing lines separate different parts of the same gel.

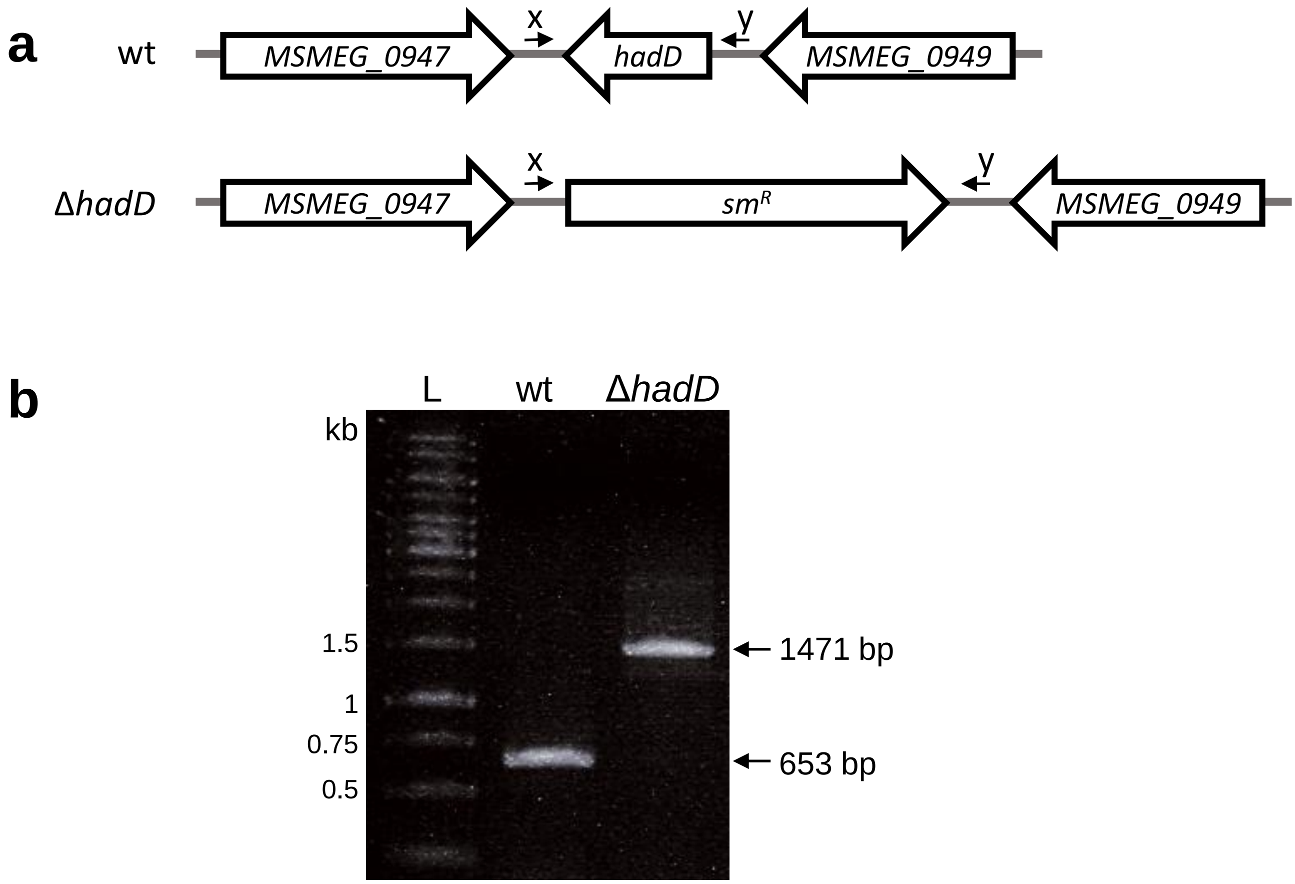

Supplementary Figure S3. Deletion of the *hadD* gene in *M. smegmatis*. (**a**) Genetic maps of the *hadD* locus in *M. smegmatis* wt and Δ*hadD* strains. The *hadD* gene has been replaced by a streptomycin resistance cassette (sm^R) *via* recombineering in the mutant strain. *MSMEG_0947* and *MSMEG_0949* gene products have unknown functions; they are annotated as "acyltransferase" and "subfamily protein IB hydrolase", respectively[2]. The primers (x and y) used for the deletion analysis by PCR (see panel b), symbolized by arrows, are located outside the *hadD* gene. (**b**) Deletion analysis by PCR. Representative agarose gel of the PCR products obtained with the x+y primer pair. Bacterial colonies of each strain were used as PCR templates. L, DNA ladder. *HadD* gene length: 534 bp; sm^R gene length: 1352 bp.

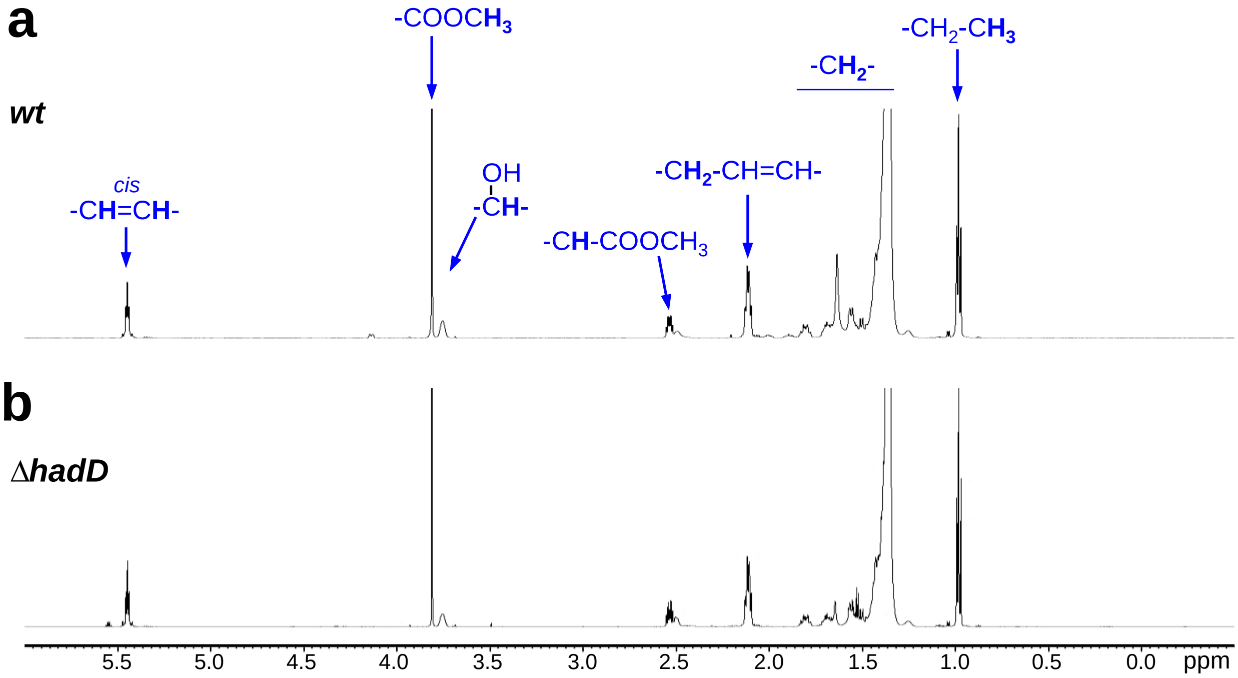

Supplementary Figure S4. The α'-mycolic acids from *M. smegmatis* wt and Δ*hadD* strains have the same structure. ^{1}H-NMR spectra of purified cell-wall-linked α'-MAs from *M. smegmatis* wt strain (**a**) and Δ*hadD* strain (**b**). Both spectra are superimposable. The proton(s) generating the signals are in bold.

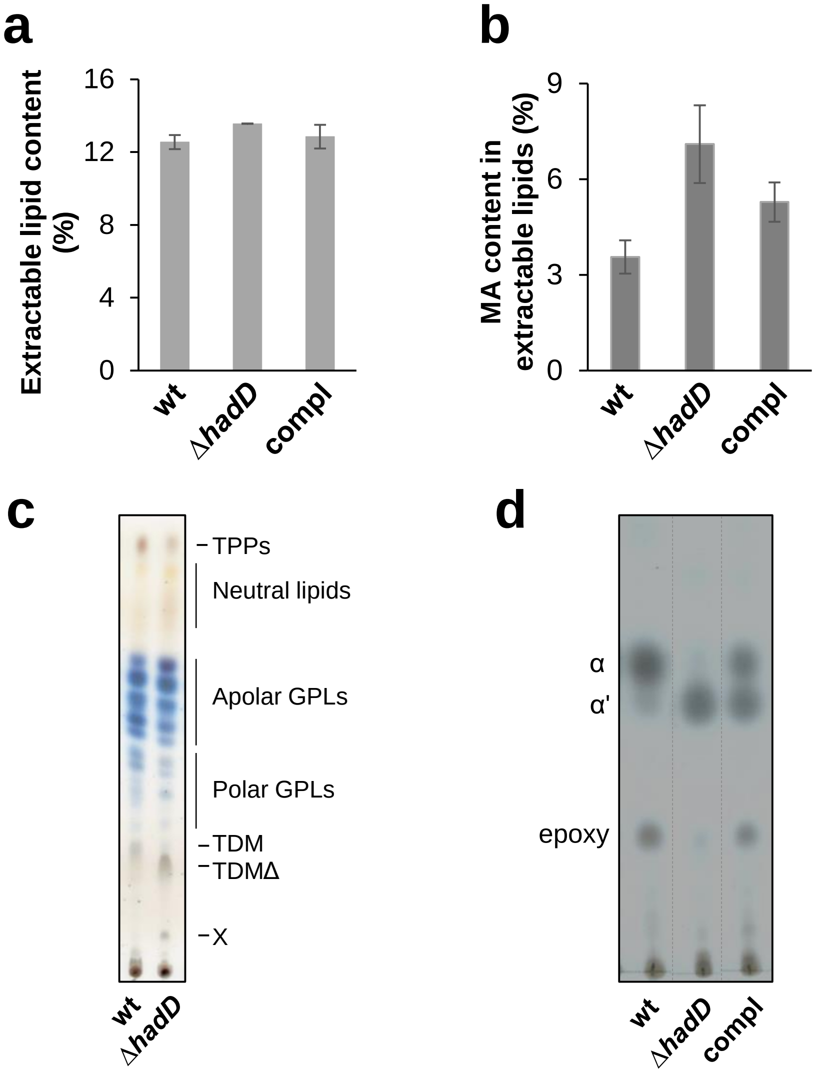

Supplementary Figure S5. Influence of *hadD* inactivation on content and mycolic acid composition of the extractable lipids. (**a**) Total extractable lipid content in the *M. smegmatis* wt, Δ*hadD* and complemented (compl) strains. (**b**) Total MA content in the extractable lipids. In (a) and (b), data are means ±average deviations. (**c**) TLC analyses of the total extractable lipids. Identical amounts of lipid mixtures were loaded on thin layer developed in $CHCl_3$:CH_3OH (9:1, v/v). Spots were revealed by anthrone spraying and heating. GPLs, glycopeptidolipids; TDM, trehalose dimycolate; TDMΔ, TDMs of *M. smegmatis* Δ*hadD*; TPPs, trehalose polyphleates; X, compound X. The apolar GPLs are diglycosylated and the polar GPLs are triglycosylated. (**d**) TLC analysis of the mycolic acyl chains (in MAME form) from the extractable lipids (TDMs and TMMs). Identical amounts of MAME mixtures were spotted on thin layer developed in dichlolomethane and revealed by $CuSO_4$ spraying and heating. Thin layers are representative of at least three independent replicates. The dividing lines separate different parts of the same thin layer.

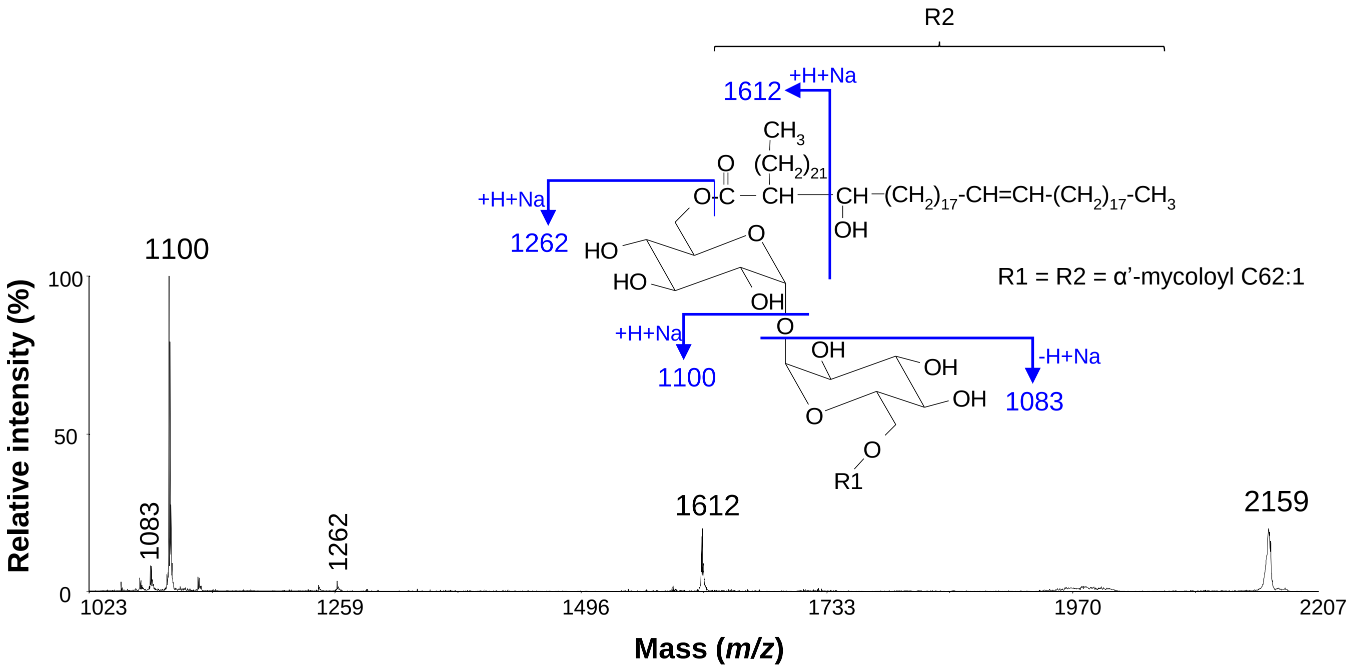

Supplementary Figure S6. MALDI-TOF/TOF MS/MS analysis of TDMΔ. Example of fragmentation of the TDM from *M. smegmatis ΔhadD* (TDMΔ) of *m/z* 2159 (see Fig. 4d).

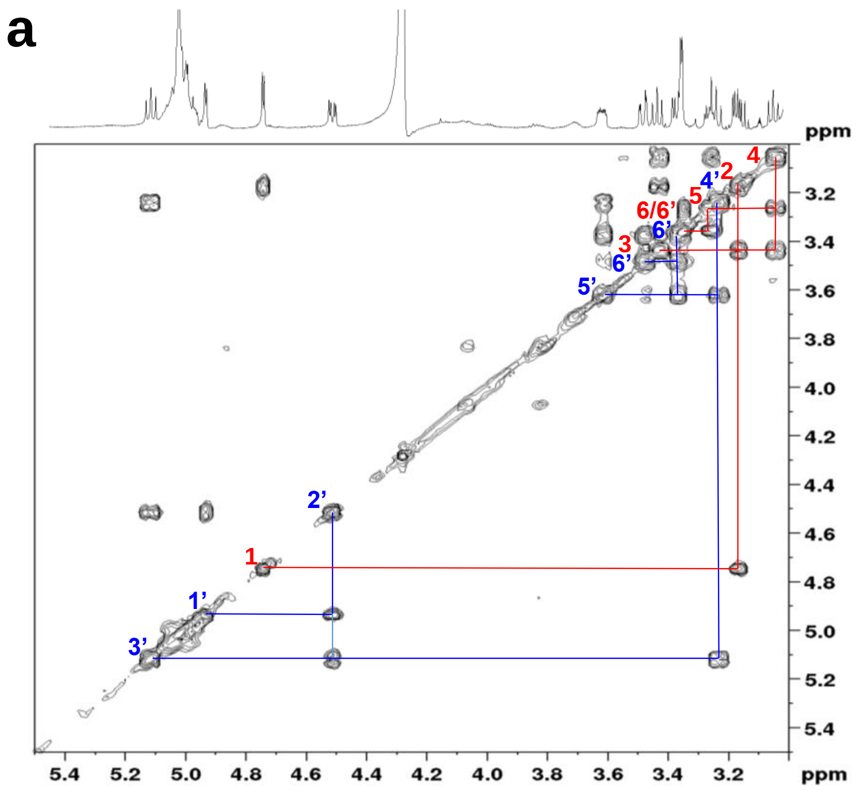

Chemical shifts

	Atom #	δ ^{1}H (ppm)
glucosyl A	1	4.78 (d)
	2	3.2 (m)
	3	3.46 (t)
	4	3.07 (t)
	5	3.29 (q)
	6a, 6b	3.37 (d)
glucosyl B	1’	4.96 (d)
	2’	4.53 (m)
	3’	5.14 (t)
	4’	3.26 (t)
	5’	3.64 (m)
	6a’	3.39 (dd)
	6b’	3.5 (dd)

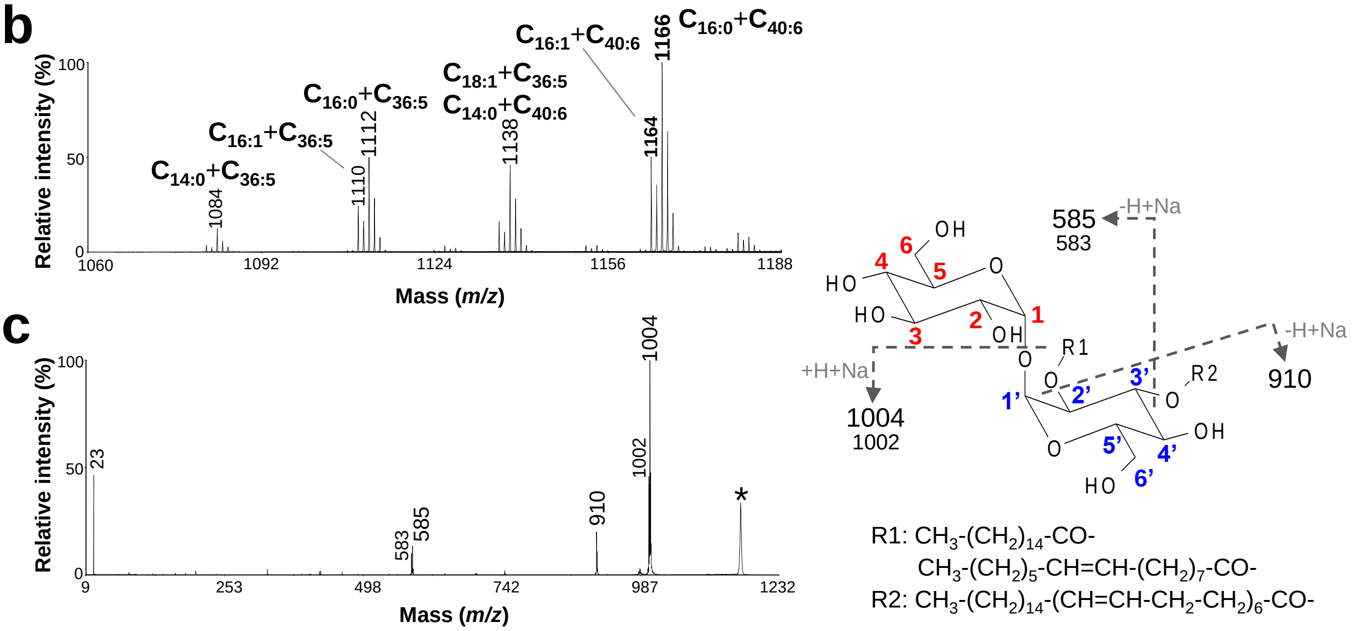

Supplementary Figure S7. Structural analyses of purified compound X. These analyses show that compound X corresponds to a mixture of 2,3-diacyl trehalose homologs, precursors of trehalose polyphleates. (**a**) Magnification of the ^{1}H-^{1}H-COSY NMR spectrum. The spin systems of the glucosyl A (red) and the glucosyl B (blue) esterified by two fatty acids are indicated. Chemical shifts are indicated in the table; multiplicities are annotated in parentheses: d, doublet; dd, doublet of doublet; t, triplet; m, multiplet. The atom numbers refer to the protons of the glucosyl residues as defined in the structure in panel (c). (**b**) MALDI-TOF MS spectrum. The major ion peaks (corresponding to sodium adducts) are labelled with their fatty acyl composition (R1+R2). The respective positions of the fatty acyl (R1) and the phleate (R2) chains may be inverted[3]. (**c**) MALDI-TOF/TOF MS/MS spectrum. Example of fragmentation of the two ion mixture of *m/z* 1164 and 1166 (see panel (b)). (*) Broad peak ranging from *m/z* 1160 to 1168, and most likely comprising both parent ions (at *m/z* 1164 and 1166). The fragment ions generated from the minor parent ion of *m/z* 1164 are labeled in smaller font. Similar fragmentations were observed for the other 2,3-diacyl trehalose homologs.

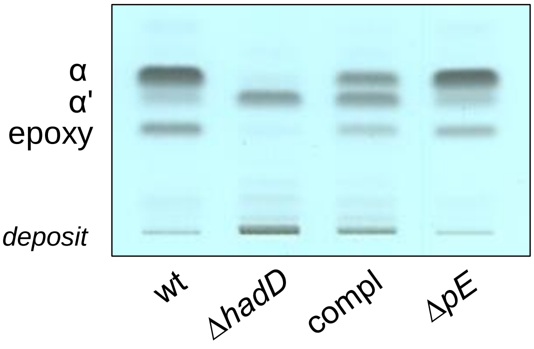

Supplementary Figure S8. The *M. smegmatis* Δ*pE* strain has a wt MA profile. HPTLC analysis of the MAMEs from *M. smegmatis* wt, Δ*hadD*, complemented (compl) and Δ*pE* strains. The thin layer was developed in dichlolomethane and stained by immersion in CuSO4 and heating. The picture is representative of three independent experiments.

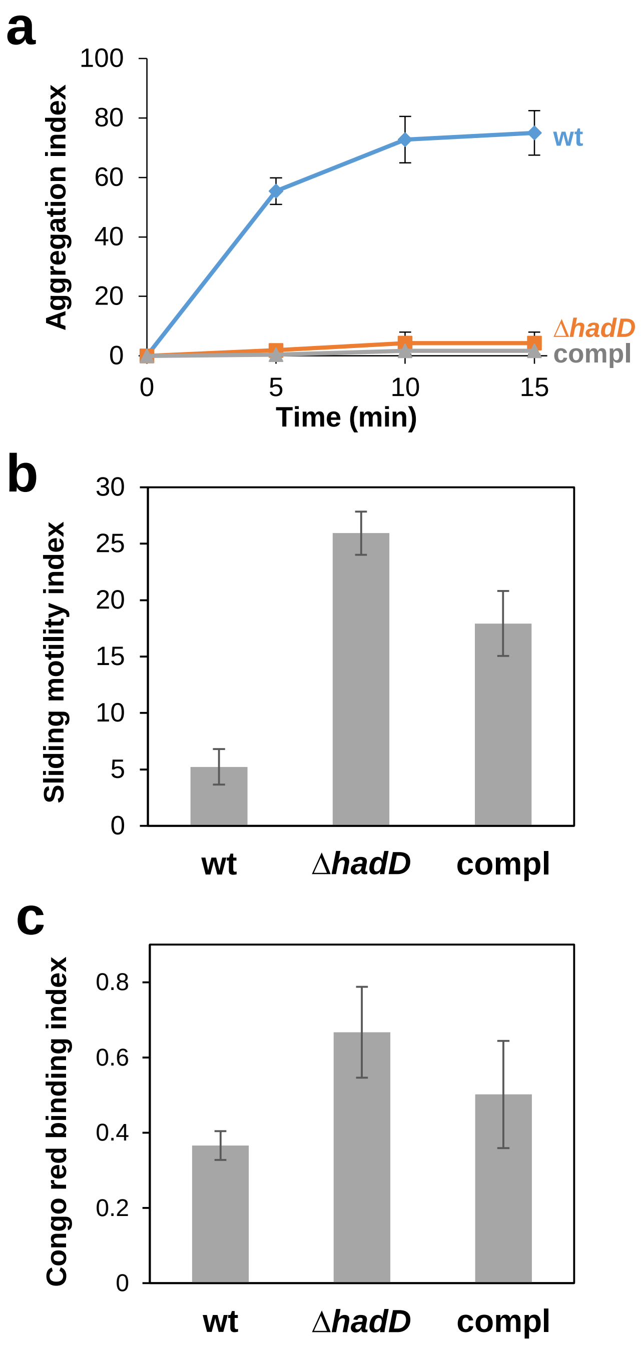

Supplementary Figure S9. Quantification of aggregation, motility and Congo red binding for the different *M. smegmatis* strains. Data are means ±average deviations. Compl: complemented strain. (**a**) Bacterial aggregation. Aggregation index as a function of time measured in 7H9 broth without Tween. The non recovery of the wt phenotype in the complemented strain might be explained by the fact that the polar GPL content remains weak (Fig. 4b) and might not reach the threshold required for effective aggregation. (**b**) Sliding motility. M63 plates solidified with 0.3% agar were inoculated from single colonies of the indicated *M. smegmatis* strains. The sliding motility index was measured after a five day incubation. (**c**) Congo red binding. The different mycobacterial strains were grown for three days in the presence of Congo red (100 µg/ml). The binding index represents the amount of bound Congo red per bacteria dry weight.

REFERENCES

1 Dong, Y. *et al.* Molecular basis for the inhibition of beta-hydroxyacyl-ACP dehydratase HadAB complex from *Mycobacterium tuberculosis* by flavonoid inhibitors. *Protein Cell* **6**, 504-517 (2015).

2 Kapopoulou, A., Lew, J. M. & Cole, S. T. The MycoBrowser portal: a comprehensive and manually annotated resource for mycobacterial genomes. *Tuberculosis* **91**, 8-13 (2011).

3 Burbaud, S. *et al.* Trehalose polyphleates are produced by a glycolipid biosynthetic pathway conserved across phylogenetically distant mycobacteria. *Cell. Chem. Biol.* **23**, 278-289 (2016).
